# Untargeted Metabolomics of *Nicotiana tabacum* Grown in United States and India Characterizes the Association of Plant Metabolomes With Natural Climate and Geography

**DOI:** 10.3389/fpls.2019.01370

**Published:** 2019-10-30

**Authors:** Dong-Ming Ma, Saiprasad V. S. Gandra, Raman Manoharlal, Christophe La Hovary, De-Yu Xie

**Affiliations:** ^1^Department of Plant and Microbial Biology, North Carolina State University, Raleigh, NC, United States; ^2^ITC Life Sciences and Technology Centre (LSTC), ITC Limited, Karnataka, Bengaluru, India

**Keywords:** climate change, continent, geography, metabolomics, nicotine, tobacco, non-polar metabolite, polar metabolite

## Abstract

Climate change and geography affect all the living organisms. To date, the effects of climate and geographical factors on plant metabolome largely remain open for worldwide and local investigations. In this study, we designed field experiments with tobacco (*Nicotiana tabacum*) in India *versus* USA and used untargeted metabolomics to understand the association of two weather factors and two different continental locations with respect to tobacco metabolism. Field research stations in Oxford, North Carolina, USA, and Rajahmundry, Andhra Pradesh India were selected to grow a commercial tobacco genotype (K326) for 2 years. Plant growth, field management, and leaf curing followed protocols standardized for tobacco cultivation. Gas chromatography–mass spectrometry based unbiased profiling annotated 171 non-polar and 225 polar metabolites from cured tobacco leaves. Principal component analysis (PCA) and hierarchical cluster analysis (HCA) showed that two growing years and two field locations played primary and secondary roles affecting metabolite profiles, respectively. PCA and Pearson analysis, which used nicotine, 11 other groups of metabolites, two locations, temperatures, and precipitation, revealed that in North Carolina, temperature changes were positively associated with the profiles of sesquiterpenes, diterpenes, and triterpenes, but negatively associated with the profiles of nicotine, organic acids of tricarboxylic acid, and sugars; in addition, precipitation was positively associated with the profiles of triterpenes. In India, temperature was positively associated with the profiles of benzenes and polycyclic aromatic hydrocarbons, but negatively associated with the profiles of amino acids and sugar. Further comparative analysis revealed that nicotine levels were affected by weather conditions, nevertheless, its trend in leaves was independent of two geographical locations and weather changes. All these findings suggested that climate and geographical variation significantly differentiated the tobacco metabolism.

## Introduction

The global warming trend has taken place over the last 100 years (Keller, 2007; Schnoor, 2007). With this continuous trend, the global mean temperature is predicted to increase by 1–4°C in the next 50–100 years (Jones, 2013; Kintisch, 2013). Accordingly, from last few decades global research efforts in different countries and regions have been undertaken to understand the effects of both global warming and regional climate changes on natural systems and human health. More endeavors are continuously being explored globally. The main research topics that have been studied and are being studied are focusing on the global scale impacts of climate change. These include effects of climate change on ecosystem and biodiversity, sea level rising, ocean acidification, water resources and desertification, agriculture and food security, and human health (Gosling et al., 2011). To date, all completed global research has led to numerous accomplishments which provide fundamental evidence that climate change significantly affects the global and regional ecosystems (Polley et al., 2013; Schmitz, 2013; Kizildeniz et al., 2015; Park et al., 2015; Hauser et al., 2016; Taylor and Kumar, 2016), agriculture, such as biotic and abiotic stresses (Adams et al., 1998; Dwivedi et al., 2013; Chauhan et al., 2014; Abberton et al., 2016; Kollah et al., 2016), and human health, such as the increase of infectious diseases and other public health problems (Patz et al., 1996; Rom and Pinkerton, 2014; Clayton et al., 2015; Kjellstrom et al., 2016). All of those data are fundamentally informative for different countries and global organizations to develop strategies and policies to slow down the global warming. To appropriately understand the effects of climate change on ecosystems, agriculture, and food safety, more endeavors are necessary to complete basic research at the molecular, biochemical, chemical, and physiological levels. However, this type of research is limited. For example, few studies have been reported to understand the effects of the combination of climate change and geography on natural product composition associated with crop and food safety and quality. Furthermore, there are particularly fewer reports regarding the comparative investigations of plant or crop metabolism associated with climate change across the continents. One of the main challenge is to conduct experiments using model plants or crops with the same genetic background that can be grown in the field in different continents. Accordingly, development of such a system is necessary to comprehensively enhance our understanding of the biological consequences of climate change.

Tobacco (*Nicotiana tabacum*) is an appropriate plant to study the effects of climate change on plant metabolism. As well characterized, metabolism in tobacco is complicated by biosynthetic networks of diverse plant metabolites such as alkaloids, terpenoids, phenolics, polyketides, alkanes, alkynes, and other metabolites, among which tobacco alkaloids, such as nicotine, cause public health problems (Sierro et al., 2014). Furthermore, more than 4,200 metabolites have been identified from tobacco plants and more than 8,000 metabolites in tobacco smoke (Rodgman and Perfetti, 2009). Currently, approximately 125 countries in Asia, Africa, North America, South America, Europe, and Australia grow tobacco plants on more than 4.3 million hectares (Eriksen et al., 2015). One primary variety grown in all these regions is the flue-cured tobacco cultivar K326, and its genome sequencing was completed a few years ago (Sierro et al., 2014). All these advantages allow K326 to be an appropriate crop plant to study the effects of climate change and geographic region or a combination of both on plant metabolism.

In this study, we used K326 tobacco to conduct a 2-year pilot study in order to understand the effects of climate change and two continental growth locations on plant metabolism. Based on our previous metabolic profiling of a small number of tobacco samples collected from tobacco companies in India, North Carolina and Brazil (Ma et al., 2013), we hypothesized that weather and growing sites in different continents differentially altered the tobacco metabolomes associated with abiotic and biotic stresses. To test this hypothesis, we selected two field locations, the Oxford tobacco research station in North Carolina, USA, and the ILTD research station (department) of ITC limited at Rajahmundry, in Andhra Pradesh, India. These two locations have been used for long-term tobacco field research. We comparatively grew K326 tobacco plants in these two locations for two consecutive years. To understand the effects of climate on plant metabolism, we followed the standard tobacco industry protocols to manage field growth, collect, and cure leaf samples. We recorded daily temperature and precipitation conditions at these two locations. A large number of samples were collected for metabolic profiling of untargeted non-polar and polar metabolites using gas chromatography–mass spectrometry (GC-MS). A metabolite analysis pipeline was developed with Mass Professional Profile (Agilent Technologies, Inc). Metabolite profiles, main weather factors, and geographic locations were integrated for association analysis. The resulting data indicated an association of metabolic variations with climatic and geographical factors.

## Materials and Methods

### Field Study Sites and Selection of Tobacco Variety

Two different continental field sites were selected for plant growth Viz., the Oxford research station in Oxford, North Carolina (latitude: 36, longitude: −78), USA and the ILTD research station of ITC limited in Rajahmundry, Andhra Pradesh, India (latitude: 16, longitude: 80). At each research station, three field plots were designed to grow plants, each plot had an area of 4047 m^2^ (one acre) and constituted one field replicate (**Supplementary Figure 1**). The variety of *N. tabacum* cultivar used was K326, which is one of the primary flue-cured tobacco crops grown globally. The rationale was that this tobacco cultivar produces a large number of plant natural products and its field growth protocol has been industrially standardized for farmers in different continents. In addition, its secondary metabolism has been well documented in the literature. Therefore, it is an appropriate crop plant to investigate the effects of climate and geography on plant metabolism.

### Experimental Design, Planting, and Sampling in North Carolina

Field growth of plants (**Supplementary Figure 1**) followed protocols documented for K326 in these two regions. The growth guide for K326 is published for farmers in North Carolina every year (https://content.ces.ncsu.edu/catalog/collection/23069/flue-cured-tobacco-information). We used this commercial growth guide to perform our experiments. Seeds were planted in soil contained in floating nursery beds in the greenhouse (**Supplementary Figure 2A**) at the end of February. This is the tobacco farming technique for commercial purpose. Seedlings were not removed out of the greenhouse until the second week of the May of 2011 (year 1) and 2012 (year 2), when plants were 72 days old. All seedlings grew strongly and uniformly (**Supplementary Figure 2B**). In North Carolina, prior to transplant, three plots were supplemented with 560 kg ha^−1^ 8-8-24 base fertilizer and treated with clomazone and carfentrazone-ethyl/sulfentrazone for weed control. Each plot was planted with 800 plants (**Supplementary Figures 2C, D**) in the second week of May 2011 and 2012 (planting date was dependent upon weather). Thus, 2,400 plants in total were grown in each farming season. Plants were later side dressed with 168 kg ha^−1^ of 15.5-0-0 fertilizer. Field growth management, such as irrigation (two to three times with irrigation reel and water from a lake) and the use of fungicides and herbicides, followed protocols documented in the research station. Plant growth was recorded and photographed each month (**Supplementary Figure 1**). When plants started to develop flower buds in early July, we selected 20 plants in each plot and then grouped 10 plants as one biological replicate. Consequently, each plot had two biological replicates. In total, 60 plants were selected from three plots and formed six biological replicates. All these plants almost grew the same height, flowered on the same date, and had 24 leaves. After this step, all plants were topped to keep 20 leaves from the bottom to the top and tagged group numbers from I to VII as shown in **Supplementary Figure 3**. After topping, plants were sprayed with maleic hydrazide to control suckers (axillary buds). After 3 weeks of topping, we started to harvest the leaf samples from group I at the bottom to group VII at the top (**Supplementary Figure 3**) on Aug. 3, Aug. 24, Sept. 14, and Oct. 4 (**Supplementary Table 1**). In total, we harvested seven groups of leaf samples, groups I–VII (**Supplementary Table 1** and **Supplementary Figure 3**). All samples were harvested in the morning. After the leaves were excised from the stems, were immediately separated into two halves, labeled as half 1 and half 2 (**Supplementary Figure 4**). The second half section included the main middle vein and was immediately cured in barn for metabolomics experiments. The first half section without the main middle vein was immediately frozen in liquid nitrogen, transported to laboratory by car, and then stored in −80°C freezers for other experiments. After 1 month of curing, 42 cured biological samples were obtained for metabolomics analysis in each year. In addition, 10 additional plants in each plot were selected and pooled as one biological sample for leaf harvest. However, leaves from these plants were not cut into two halves. Accordingly, seven uncut groups (I–VII, **Supplementary Figure 3**) of leaves from 30 plants were harvested and immediately cured in the same barn. In total, 21 uncut biological samples were obtained (**Supplementary Table 2**). These samples were used as control to evaluate if cutting leaves into two halves could affect metabolite profiles. In summary, 84 cut leaf (biological) samples were collected from seven leaf positions (**Supplementary Figure 3**, **Supplementary Table 1**) and cured for metabolic profiling. To simplify the sample name description for data analysis, “U” was used to represent USA, and “F” and “S” were used to represent the first and second year of growth. Based on positions defined for sampling (**Supplementary Figure 3**), we used U-F1H through U-F7H to describe USA’s samples in the first year from positions 1 to 7 harvest. We also used U-S1H through U-S7H to describe USA’s samples in the second year from positions 1 to 7 Harvest. Each group of samples was composed of six biological replicates (**Supplementary Tables 1** and **3**). In addition, 42 entire leaf (un-cut, biological) samples as control were collected from seven positions (**Supplementary Table 2**) and cured for control metabolic profiling. Cured samples were ground into fine powder. For each biological sample, three technical replicates were prepared for non-polar and polar metabolite extraction described below.

### Experimental Design, Planting, and Sampling in India

In India, field design, experimental design, and field management practices were the same as described above. However, field growth of plants and sampling times (**Supplementary Table 3**) were different from those in North Carolina because of tropical weather in India. In addition, plants were irrigated 6–8 times with ground water using a furrow irrigation method.

Moreover, the protocol developed for farmers requires to keep 22 leaves in each plant (**Supplementary Figure 5**). Therefore, in our experiments, after topping, each plant had 22 leaves, which were grouped into eight groups (**Supplementary Figure 5**). The leaf sampling approach was the same as described above for North Carolina’s samples (**Supplementary Figure 4**). In total, 48 biological leaf samples with a main vein (midrib) (**Supplementary Table 4**) were collected and cured for metabolomics in each year. Samples frozen in liquid nitrogen were transported to ITC laboratory by air flight and then stored in −80°C freezers. In addition, 24 biological samples of entire leaves without cutting were harvested and then cured as control for metabolomics in each year. Daily weather conditions during two growing seasons were obtained from local weather station. In summary, in 2 years of growth, 96 cut leaf (biological) samples were collected from eight positions (**Supplementary Table 3**) and cured for metabolomics. To simplify the sample’s name description used for data analysis, we used “I” to represent India (**Supplementary Tables 3**). We used I-F1H through I-F8H to describe India’s samples in the first year from positions 1 to 8 harvest. We also used I-S1H through I-S8H to describe India’s samples in the second year from positions 1 to 8 harvest. Each group of samples included six biological replicates (**Supplementary Tables 3**). In addition, 48 entire leaf (un-cut, biological) samples were collected from eight positions (**Supplementary Table 4**) and cured as control. Cured samples were ground into fine powder. Powdered samples were then shipped to North Carolina State University for metabolic profiling. For each biological sample, three technical replicates were prepared for non-polar and polar metabolite extraction described below.

### Extraction of Non-Polar Metabolites

Extraction of non-polar metabolites was performed as reported previously (Ma et al., 2013). In brief, 100 mg of powdered sample was suspended in 1.5 ml 100% hexane in a 2 ml Eppendorf tube. The extraction tube was vortexed vigorously for 1 min, followed by 30 min sonication, and then placed in a 56°C water bath for additional 50 min. The extraction tube was centrifuged at 11,000 rpm for 10 min. The resulting clean hexane supernatant was pipetted into a new clean tube. A 200 µl hexane extract was pipetted into a 400 µl insert contained in a 2 ml glass vial (Agilent) for gas chromatograph-mass spectrometry analysis described below.

### Extraction of Polar Metabolites and Derivatization

One hundred milligram of powdered sample was suspended in 1.5 ml extraction solvent (methanol: chloroform: water, 5:2:2) in a 2.0 ml Eppendorf tube and then vigorously vortexed for one min. The extraction tube was sonicated for 30 min in water bath and placed in a 56°C water bath for 50 min. The extraction tube was centrifuged at 11,000 rpm for 10 min. One milligram of the supernatant was transferred to a new clean 1.5 ml tube, to which 300 µl chloroform and 600 µl double deionized H_2_O were added. The tube was vigorously vortexed for 1 min to mix solvents thoroughly, followed by centrifugation at 4,000 rpm for 5 min to result in the upper water-methanol (polar) phase and the lower chloroform (non-polar) phase. One hundred microliter water-methanol phase was transferred to a new 1.5 ml Eppendorf tube and then was evaporated in a rotary vacuum at room temperature. The remaining residues at the bottom of tubes contained polar metabolites, which were used for derivatization as reported previously (Ma et al., 2013). In brief, the remaining residue was oximated using 40 µl methoxylamine hydrochloride (20 mg/ml) in anhydrous pyridine at 37°C for 2 h, and then were silylated at 37°C for 30 min using 70 µl of N-methyl-N-(trimethylsilyl)-trifluoro acetamide (MSTFA). The liquid derivatized samples were pipetted into 400 µl glass vials for GC–MS analysis as reported (Duan et al., 2012), which is described below.

### Gas Chromatograph-Mass Spectrometry Analysis

An Agilent 6890 Gas Chromatograph coupled with 5975 MSD (Agilent Technologies, USA) was used to profile metabolites extracted from 180 cut leaf samples (84 from North Carolina and 96 from India) and 90 (42 from North Carolina and 48 from India) uncut entire leaf samples (**Supplementary Tables 1**–**4**). Prior to injection of samples, we carefully performed instrumental quality assurance and quality control (QA/QC), including but not limiting to the use of new consumables, cleaning of systems, tuning, blank solvent control, and others. Three technical replicates were prepared for each biological sample, of which two technical replicates were analyzed. The third technical replicate was assayed for some of samples when it was necessary. As a result, 12 or 18 repetitive assays for each group leaf sample (such as U-F1H from group I, **Supplementary Figures 3** and **4**) were carried out to profile non-polar and polar metabolites. A RTX-5 capillary column (30 m×0.25 mm×0.25 µm) was used to separate metabolites. The inlet was operated using a splitless mode. One microliter sample was injected to profile metabolites. The injection temperature was set at 250°C. The temperature of column oven was initially set at 60°C for 2 min, then ramped to 320°C at a constant rate of 8°C/min, and held at 320°C for 2 min. Pure helium was used as the carrier gas with a flow rate of 1 ml/min. A positive electron impact ion source (70 EV) was used to ionize compounds and mass fragments were scanned in the range of 40–800 (m/z) starting with 4 min of retention time. We recorded total ion chromatographs for all samples. In addition, a mixture of even-numbered chain n-alkenes (C10–C40) purchased from Restek (Florida, USA, catalog no. 31266) were used to estimate the retention index values, which were used to deconvolute metabolite peaks as reported previously (Almaarri et al., 2010).

### Data Processing and Statistical Analysis

GC-MS ChemStation data format for metabolites detected from each assay was translated into the MassHunter data format using Agilent MassHunter GC/MS Translator Software (version B.05.02). Then, untargeted analysis was carried out using the Mass Hunter Qualitative Analysis software (Version B.06.00) for metabolite deconvolution, compound search, and identification in NIST11 library. When the MS profile of an untargeted metabolite showed no less than 80% identity to that of a standard metabolite in the library, it was annotated to the standard. Then, the MassHunter data format of all annotated metabolites was exported as Compound Exchange Format (.cef). All “cef” files were imported to the Mass Profiler Professional (MPP, version B.12.5, Agilent) for statistical analysis.

The resulting “cef “ files for all samples were imported to MPP for principal component analysis (PCA) and hierarchical clustering analysis (HCA) according to our previous report (Ma et al., 2015). For PCA, we used Eigen-vector based scaling method to perform alignment and normalization (using log2) and then visualize metabolite profiles in all samples. The abundance of metabolites was evaluated using counts. In MPP, the minimum abundance count and retention time tolerance (min) values across all sample sets were 5,000 and 0.05, respectively. These threshold values were used as a filter to determine the presence or absence of metabolites in samples. In addition, HCA is a statistical method to group samples that are unsupervised in different clusters or branches of the hierarchical tree. The MPP software processing tool is effective to align all the data and then normalize them using log 2 for each metabolite. The MPP identified the median value across all the biological samples (180 cut leaf samples) and then baselined each metabolite account value to the median value to establish the hierarchical clustering (conditional tree) and heat maps. The resulting tree showed the relationships between different groups of samples from both North Carolina and India.

### Pearson Analysis and Principal Component Analysis for Metabolites, Temperature and Precipitation in North Carolina and India Alone

Pearson analysis was performed using the SPSS software (IBM SPSS Statistics 22) to understand the potential relevance of one or two climate factors and metabolite profiles of leaves from in both India and North Carolina. Weather stations in Oxford County in North Carolina, USA, and Rajahmundry in Andhra Pradesh, India recorded local field air temperature and daily precipitation. We obtained these data from weather stations and then calculated the average values for every week of plant growth. To perform Pearson analyses, variables that were used included average temperature and precipitation values in each harvest week, nicotine (a main tobacco alkaloid in leaves) peak values, and peak values of 11 groups of metabolites (including six groups of non-polar and five groups of polar metabolites, **Supplementary Table 9**). The six non-polar metabolite groups included monoterpenes, sesquiterpenes, diterpenes, triterpenes, benzene, and polycyclic aromatic hydrocarbons (PAH). The five polar metabolite groups included sugars, amino acids, organic acid, nitrogen-containing secondary metabolites (nicotine-related compounds) and polyphenol. The level of each group resulted from the sum of peak values of all individual metabolites. For example, the monoterpene level was summed from all individual molecule peak values. The resulting average temperature values per week, average precipitation values per week, and total peak values of each group of metabolites were input to the SPSS software for Pearson analysis.

Eigen-vector based PCA was carried out with the JUMpro 12 software (North Carolina State University, Raleigh, USA) to understand the potential relevance between 12 groups of metabolites (nicotine and 11 groups), average daily temperature, and average daily precipitation at locations in both India and North Carolina. The PCA type used during the computation was the Pearson’s correlation matrix. This analysis corresponds to the classical correlation coefficiency. The resulting plots were characterized by arrows that represented observations and variables simultaneously. When two variables were distant from the plot center, there were three results. The first is that two are close to each other, indicating that they were significantly positively correlated (r close to 1). The second is that two are orthogonal each other, indicating that they are not correlated (r close to 0). The third is that two are opposite to each to other, indicating that they are significantly negatively correlated (r close to −1).

## Results

### Non-Polar Metabolite Profile Complexity in Leaves From North Carolina and India

We used GC-MS to complete 387 assays for 180 biological samples. These included 84 and 96 cut leaf (biological) samples collected from 2 years’ field growth studies in North Carolina and India, respectively. The 84 biological samples from North Carolina were composed of seven groups of leaves and the 96 biological samples from India were composed of eight groups of leaves. Each group of samples was composed of six biological replicates. Two technical replicates for each biological sample were analyzed using GC-MS. In addition, 27 biological samples were randomly selected from 180 samples for a third technical replicate analysis in order to confirm experimental reproducibility of GC-MS. As a result, GC-MS analysis detected more than 700 different metabolite peaks from 387 extractions (**Supplementary Table 5**). When a peak was detected from at least 20 samples, it was considered being a positive one. The peak was then annotated as a compound with at least 80% identity to a standard in the library. Accordingly, 171 non-polar metabolites (**Supplementary Table 6**) were annotated from 387 assays. Based on skeleton structure features, these metabolites were classified into nine groups, including: linear hydrocarbons, cyclic hydrocarbons, alcohol, aldehyde, ketones, acids, terpene, polyphenol, and nitrogen-containing natural products.

**Figure 1:**
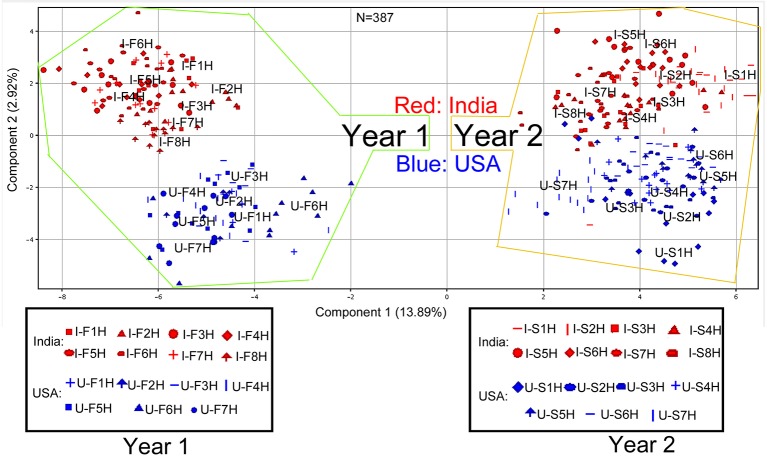
Scaling principle component analysis (PCA) for two consecutive years’ nonpolar metabolomes from leaves of *Nicotiana tabacum* K326 grown in the field of India and North Carolina. This analysis was completed with MPP software. In total, 387 extracted samples were analyzed with gas chromatography–mass spectrometry. MPP was used to annotate 171 nonpolar metabolites. All samples and annotated metabolites were used for PCA. Each data point represents a linear combination of 171 metabolites from a single extract. Red color represents samples from India and blue color represents samples from USA. Samples were labeled by their abbreviations. I-F1H through F8H are India’s samples in the first year from positions 1 to 8 harvest. U-F1H through F7H are USA’s samples in the first year from positions 1 to 7 harvest. I-S1H through S8H are India’s samples in the second year from positions 1 to 8 harvest. U-S1H through S7 are USA’s samples in the second year from positions 1 to 7 harvest; N = 387: 387 extracted samples.

All 171 metabolites annotated and their ion chromatographic peak values, growing years, and locations were used as entries for PCA in MPP. The resulting two-dimensional ordination plot showed that the first principal component (PC1) and the second principal component (PC2) accounted for 13.89 and 2.92% of the total variance of metabolites across 387 extracted samples from two different years across two growth locations (**Figure 1**). Based on this plot, the ordination orders of two growing years and locations were separated by variables consisting of 171 metabolite and 387 extracted samples. Regardless of the locations, the growing years were ordinately separated in the PC1 axis, indicating differential effects of growing years on the profiles of 171 metabolites. In each year, North Carolina (USA) and India were ordinately separated in the PC2 axis, indicating the difference of metabolism of these 171 metabolites in two growing locations.

**Figure 2:**
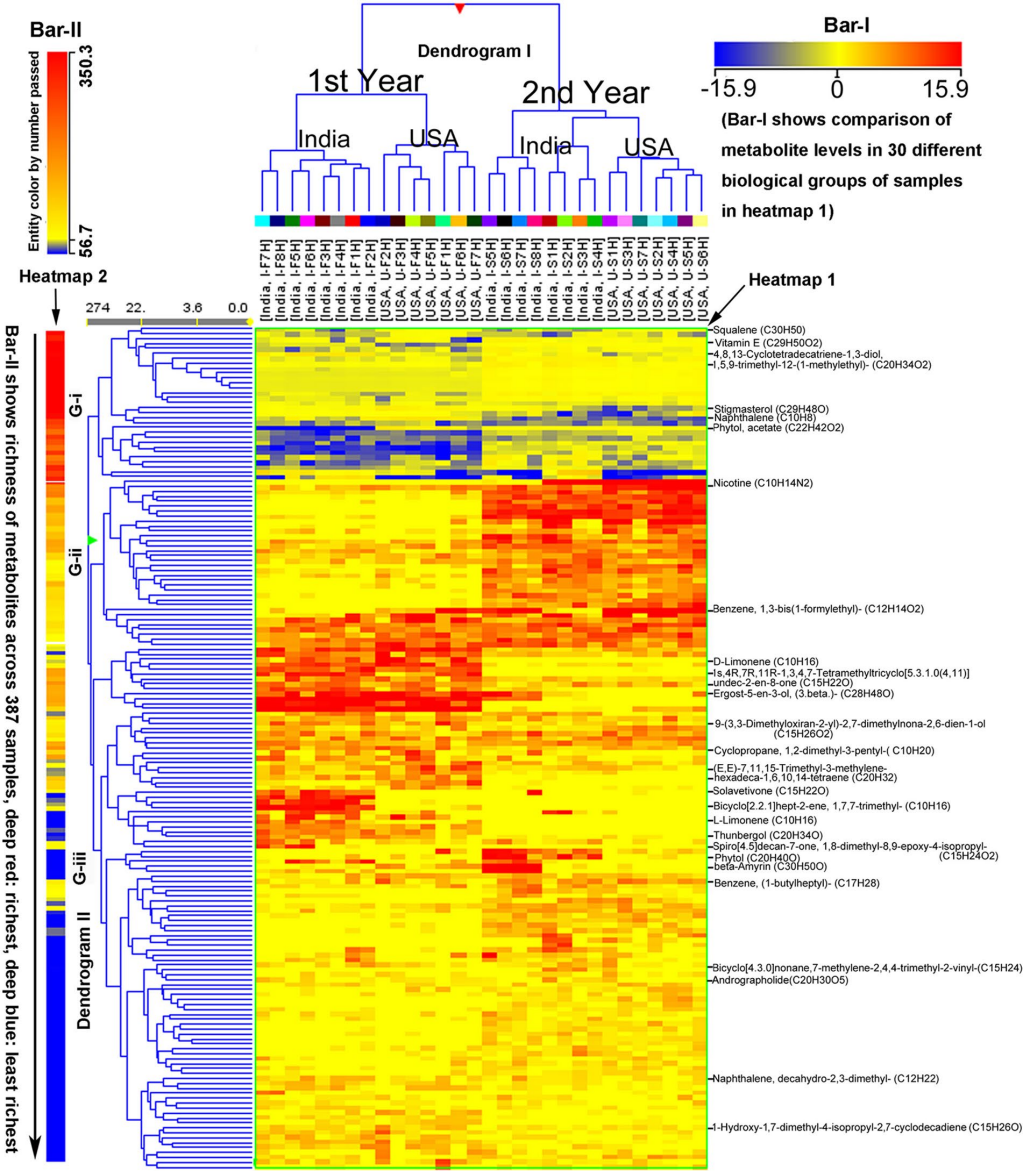
Hierarchical cluster analysis (HCA) for two consecutive years’ nonpolar metabolomes from leaves of *Nicotiana tabacum* K326 grown in the field of India and North Carolina. A heat map resulted from HCA using log2-fold changes of 171 metabolites during two growing seasons in India and USA. Fold changes and color (bar-I) resulted from normalization that was carried out using each log2 value of each metabolite peak value in each sample to compare its median peak value across all 387 extracted samples. Two color bars are used in the heat map. The horizontal bar-I fold color bar (15.9 to −15.9) from deep red to deep blue shows high to low levels of each metabolite in different groups of biological samples. The bar-II entity color by number passed (350.3–56.7) and the long arrow from the top to the bottom featured using deep red to deep blue color indicate the frequency of metabolites detected from the highest (deep red) to the lowest (deep blue) richness in all 387 extracted samples. For example, squalene listed in the top of the heat map was the most abundant metabolite detected in all samples, while naphthalene, decahydro-2,3-dimethyl- listed in the relative bottom with deep blue color was one of the least abundant metabolite detected in all samples. Samples were labeled by their abbreviations. I-F1H through F8H are India’s samples in the first year from positions 1 to 8 harvest. U-F1H through F7H are USA’s samples in the first year from positions 1 to 7 harvest. I-S1H through S8H are India’s samples in the second year from positions 1 to 8 harvest. U-S1H through S7 are USA’s samples in the second year from positions 1 to 7 harvest; N = 387: 387 extracted samples.

The abovementioned 171 metabolites and their ion chromatographic peak values, growing years, and locations were also used for HCA. As a result, HCA created two dendrograms (I and II), two heat maps (1 and 2), and two color bars (I and II) (**Figure 2**). These graphic features allowed visualizing the clustering distance between paired samples in two growth places, e.g., I-F1H *vs*. U-F1H, and in 2 years, e.g., I-F1H *vs*. I-S1H. In addition, these graphs the revealed clustering distance between sample groups from the bottom to the top of plants, e.g., I-F1H *vs*. I-F2H. These data showed effects of two growing years and two locations on 171 metabolite profiles from 387 extracted samples (30 groups of samples). The color bar I, heat map I, and dendrogram I characterized that based on metabolite profiles, samples in each of the first and second years alone was clustered, respectively, regardless of the field location in India and North Carolina. These data were consistent with the results of PCA described above. These data also indicate that the growing years affect the non-polar metabolite complexity in plants. The heat map I and dendrogram I also showed that under each year, samples from India and samples from North Carolina were grouped together, respectively. These results indicate that in the same year, the non-polar metabolite complexity in samples depend upon each growth location. The color bar II, heat map II, and dendrogram II categorize metabolites from high to low abundance in all samples regardless of growing locations and years. The color bar II from deep red to deep blue characterizes the peak abundance of the 171 metabolites shown in heat map II, each of which is coded by different colors in the direction of the black arrow (**Figure 2**). Based on color codes, we categorized the 171 metabolites into three groups (G-i, ii, and iii). The G-i is highly abundant in all 387 extracted samples regardless of growing years and locations. Example metabolites of this group includes squalene, vitamin E, stigmasterol, some diterpenes [such as 4,8,13-cyclotetradecatriene-1,3-diol,4,8,13-cyclo tetradecatriene-1,3-diol, 1,5,9-trimethyl-12-(1-methylethyl)-(C_22_H_34_O_2_), and phytol acetate]. The G-ii coded by pink to yellow-brownish color in the clustering are middle abundant metabolites, including nicotine and 1,3-bis(1-formylethyl) benzene. The G-iii coded by yellowish to bluish color are low abundant metabolites. Examples include monoterpenes (such as limonene, 1,7,7-trimethyl-bicyclo[2.2.1]hept-2-ene, and 1,2-dimethyl-3-pentyl-cyclopropane), sesquiterpenes [such as, solavetivone, 9-(3,3-dimethyloxiran-2-yl)-2,7-dimethylnona-2,6-dien-1-ol, 1S,4R,7R,11R-1,3,4,7-tetramethyltricyclo(5.3.1.0{4,11})undec-2-en-8-one], diterpenes (such as thunbergol, andrographolide, and others), triterpenes, 1-butylheptyl-benzene, and decahydro-2,3-dimethyl-naphthalene.

### Polar Metabolite Profile Complexity in Leaves From North Carolina and India

We used GC-MS to complete total 366 assays for 180 biological samples to analyze polar metabolites. Two technical replicates were analyzed for each biological replicate. In addition, six biological samples were randomly selected from 180 samples for a third technical replicate to examine technical reproducibility. More than 500 metabolite peaks were detected from 366 assays (**Supplementary Table 7**). Based on 80% identity criteria, 225 peaks (**Supplementary Table 8**) were annotated to metabolites using the MPP software. These compounds were categorized into seven different groups, including sugar, amino acid, organic acid, alcohol and ketone, N-containing natural products, hydrocarbons, and phenylpropanoids (**Supplementary Table 8**).

**Figure 3:**
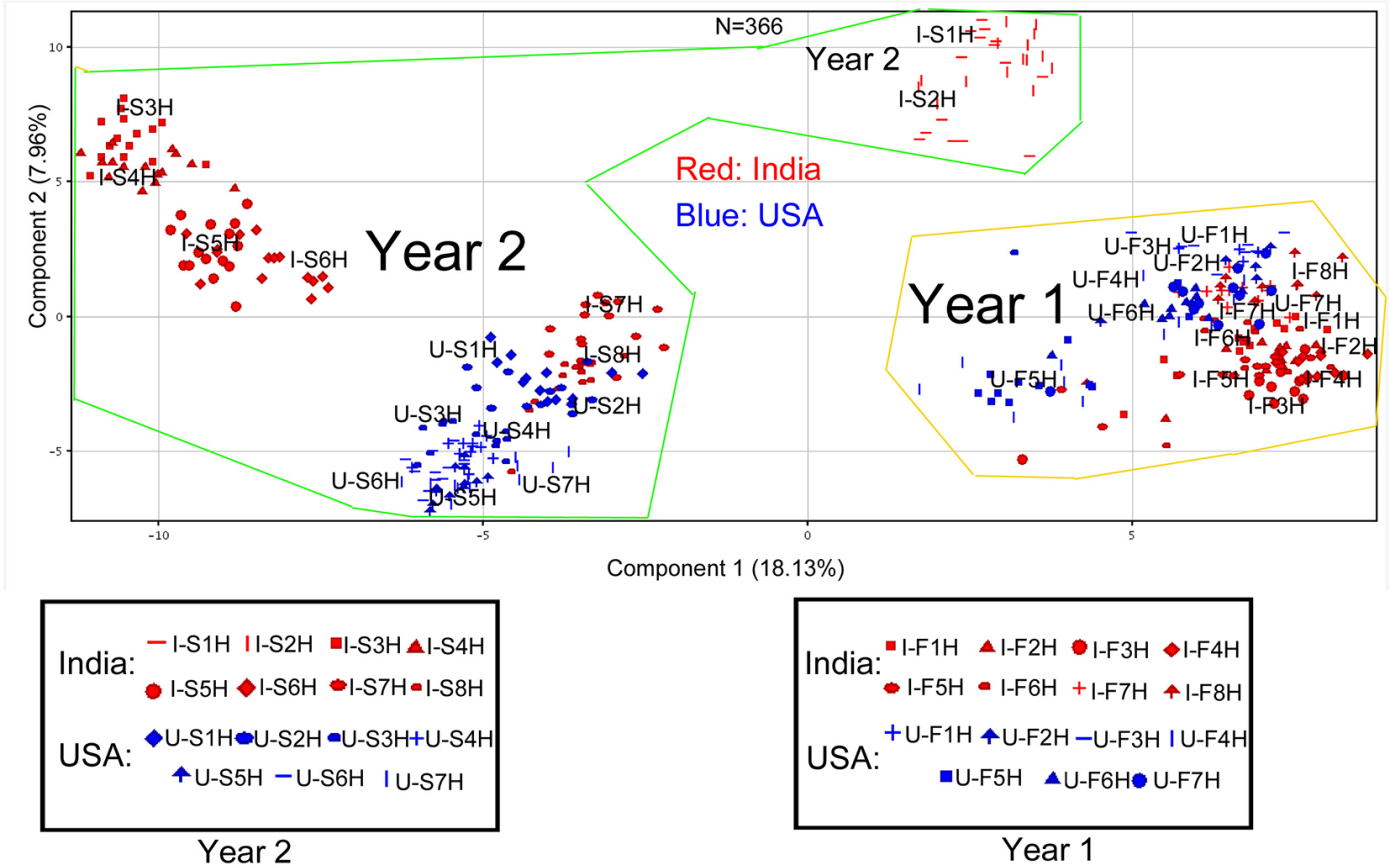
Scaling principle component analysis (PCA) for two consecutive years’ polar metabolomes from leaves of *Nicotiana tabacum* K326 grown in the field of India and North Carolina. This analysis was completed with MPP software. In total, 366 extracted samples were analyzed by gas chromatography–mass spectrometry. MPP was performed to annotate 225 peaks to polar metabolites. All samples and annotated metabolites were used for PCA. Each data point represents a linear combination of 225 metabolites from a single extract. Samples were labeled by their abbreviations. I-F1H through F8H are India’s samples in the first year from positions 1 to 8 harvest. U-F1H through F7H are USA’s samples in the first year from positions 1 to 7 harvest. I-S1H through S8H are India’s samples in the second year from positions 1 to 8 harvest. U-S1H through S7 are USA’s samples in the second year from positions 1 to 7 harvest. N = 366: 366 extracted samples.

All 225 metabolites annotated and their ion chromatographic peak values, 366 extracted samples, two growing years, and two locations were used as variables for PCA. The resulting ordination plot showed that the percentages of PC1 and PC2 were 18.13 and 7.96% across all variables (**Figure 3**). Regardless of growth locations, the resulting plot showed that the polar metabolome of the 1^st^ year’s samples was ordinately separated from that of the 2^nd^ year’s samples in the PC1 axis (**Figure 3**), indicating that growing years obviously altered the plant metabolism. In addition, the resulting plot revealed that samples from India and USA were ordinately separated in the PC2 axis (**Figure 3**), indicating different metabolism of these compounds in two growing locations. Based on this plot, the profiles of these 225 metabolites between North Carolina and India were more similar in the first year’s leaf samples (U-F1H to U-F7H and I-F1H to I-F8H) than in the 2^nd^ year’s leaf samples (U-S1H to U-S7H and I-S1H to I-S8H). It was interesting that in the second year, dynamic metabolite profile distributions were observed between North Carolina and India samples. In the 2^nd^ year, metabolite profiles in seven groups of samples (U-S1H to U-S7H) from North Carolina were closely grouped together, while the profiles of these metabolites in eight groups of samples from India (I-S1H to I-S8H) were ordinately separated into three sections in the PC1 axis (**Figure 3**).

**Figure 4:**
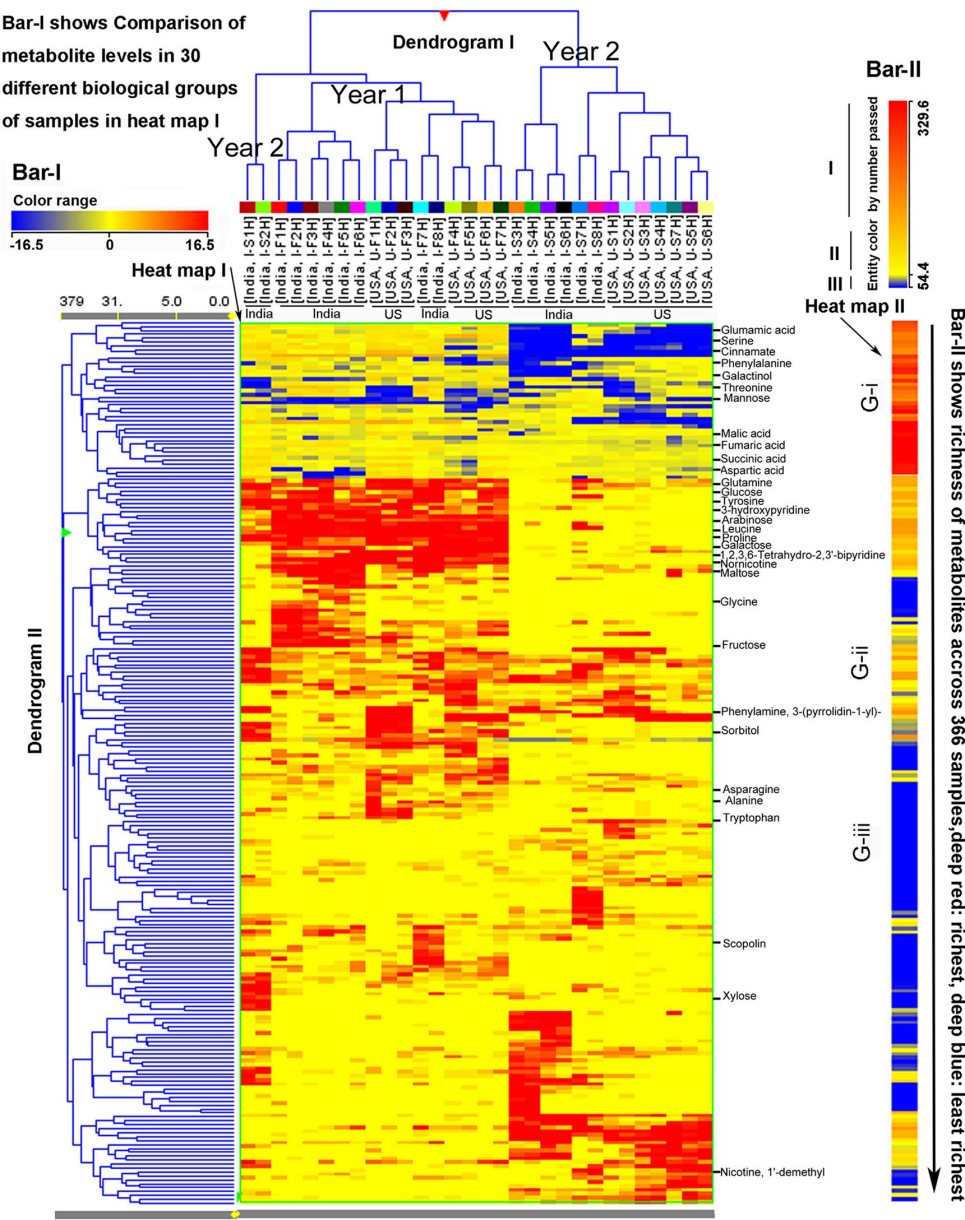
Hierarchical cluster analysis (HCA) for two consecutive years’ polar metabolomes from leaves of *Nicotiana tabacum* K36 grown in the field of India and North Carolina. A heat map resulted from HCA using log2-fold changes of 225 metabolites during two growing seasons in India and USA. Fold changes and color (bar-I) resulted from normalization that was carried out using each log2 value of each metabolite peak value in each sample to compare its median peak value across all 366 extracted samples. Two color bars, bar-I and bar-II, are used in the heat map. The horizontal bar-I fold color bar (16.5 to −16.5) from deep red to deep blue shows high to low levels of each metabolite in different groups of biological samples. The bar-II entity color by number passed (329.6–54.4) and the long arrow from the top to the bottom featured using deep red to deep blue color indicate the frequency of metabolites detected from the highest (deep red) to the lowest (deep blue) richness in all 366 extracted samples. For example, glutamic acid listed in the top of the heat map was the most abundant metabolite detected in all samples, while xylose listed in the relative bottom with deep blue color was one of the least abundant metabolite detected in all samples. Samples were labeled by their abbreviations. I-F1H through F8H are India’s samples in the first year from positions 1 to 8 harvest. U-F1H through F7H are USA’s samples in the first year from positions 1 to 7 harvest. I-S1H through S8H are India’s samples in the second year from positions 1 to 8 harvest. U-S1H through S7 are USA’s samples in the second year from positions 1 to 7 harvest. N = 366: 366 extracted samples.

The abovementioned 225 metabolites and their peak values, 366 extracted samples, two growing years, and two locations, were also used as variables for HCA. As described above, HCA created two heat maps (I and II), two dendrograms (I and II), and two color bars (I and II) to visualize metabolite profiles in 366 extracted samples (**Figure 4**). Bar I, heat map I, and dendrogram I characterized metabolite profiles in 366 extracted samples (30 groups of samples) from 2 years. The heat map I and dendrogram I show that 15 groups of the first year’s samples (eight from both India and seven from North Carolina) are clustered together, while 13 groups of the second year’s samples (seven from both North Carolina and six from India) are clustered together. Two groups of samples in the second year, I-S1H and I-S2H from India, are clustered as an out-group, which is clustered together with the first years’ samples. These results were in agreement with those obtained from PCA, in which these two groups of samples were relatively ordinately distant from 13 sample groups of the second year in the first PC1 axis (**Figure 3**). These results indicate that metabolic accumulation of plants are associated with the two growing years. Based on the tree in dendrogram I, in each year, samples from India and North Carolina were separately clustered together. These results indicate that in the same year, growth locations control metabolic complexities. Bar II, heat map II, and dendrogram II characterized peak abundance profiles of the 225 metabolites in 366 extracted samples regardless of years and locations. Based on color codes, we categorize the 225 metabolites into three groups, G-i: high abundance, G-ii: middle abundance, and G-iii: low abundance, which are colored by deep red to orange, orange to yellow, and blue, respectively (**Figure 4**). Examples of G-i include glutamic acid, malic acid, fumaric acid, succinic acid, aspartic acid, serine, phenylalanine, threonine, and others. Examples of G-ii include glucose, arabinose, fructose, and others. Examples of G-iii include xylose, 1’-demethyl nicotine, and other compounds (**Figure 4**).

### Association of Two Climatic Factors and 12 Groups of Metabolites in North Carolina

**Figure 5:**
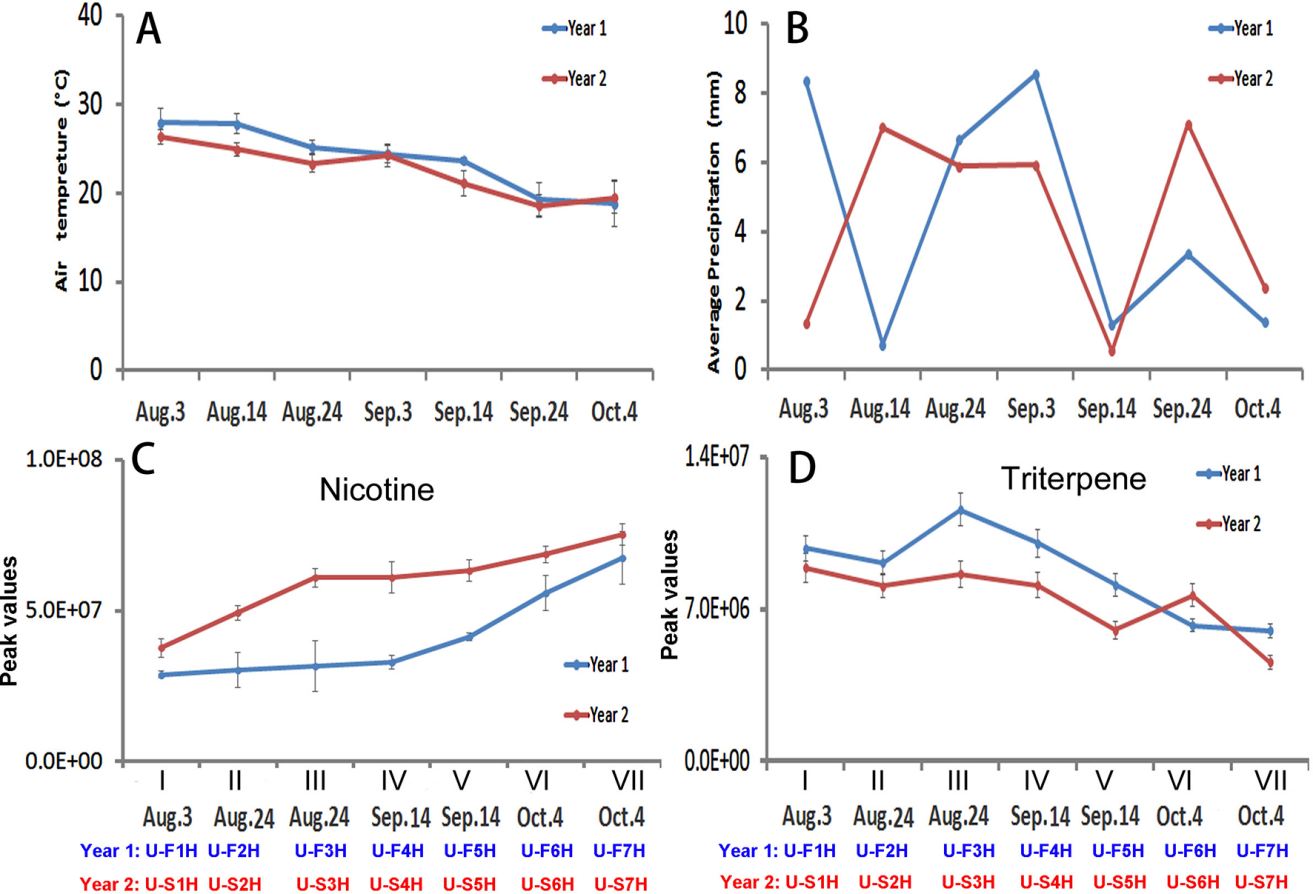
Dynamics of two climate factors and trends of two groups of metabolites in two harvesting seasons in North Carolina. **(A–B)**, Dynamics of average temperature **(A)** and precipitation **(B)** values at seven harvesting dates. Each value resulted from averaging daily temperature or precipitation levels in that week of harvest. (C-D) Trends of total nicotine levels **(C)** triterpene levels **(D)** in seven groups of samples harvested on the four harvesting dates. I, II, III, IV, V, VI, and VII: seven groups of samples, in which II and III were collected on Aug. 24, IV and V were collected on Sept. 14, and VI and VII were collected on Oct. 4.

Both air temperature and precipitation of two growing seasons in North Carolina were recorded daily. Average values for each leaf harvest week were calculated. The dynamic trend of average temperature values was similar at Oxford research station in 2 years. The values decreased from the first (Aug. 3) to the last (Oct. 4) harvest week (**Figure 5A**). The average temperature values were higher in the 1^st^ (Aug. 3), 2^nd^ (Aug. 14), 3^rd^ (Aug. 24) and 5^th^ (Sept. 14) weeks of harvest in the 1^st^ year than in the 2^nd^ year (**Figure 5A**). The dynamics of average weekly precipitation values were different in the 2 years of growth (**Figure 5B**). During the seven continuous weeks of harvest, the average precipitation values in the 3^rd^ (Aug. 24), 5^th^ (Sep. 14) and 7^th^ (Oct. 4) were relatively close, but were higher in the 1^st^ and 4^th^, and lower in the 2^nd^ and 6^th^ week of harvest in the first year than in the second year.

The level trends of nicotine levels and 11 groups of metabolites (triterpenes, monoterpenes, diterpenes, sesquiterpenes, benzene, PAH, amino acid, organic acids of the tricarboxylic acid (TCA) cycle, sugars, nitrogen containing compounds, and polyphenols) in seven groups of leaves were characterized using their peak values (**Figures 5C, D**; **Supplementary Figure 6**). Except for nicotine, the levels of each group of metabolites were summed from individuals, such as the level of monoterpenes summed from all individuals. The resulting plots showed three types of trends in 2 years. The first trend was similar in 2 years but the levels of group metabolites at each harvest date were lower in the first year than in the second year. This type included nicotine and benzenes. The trends of nicotine in 2 years of growth were similar from the lowest level in the group I leaves to the highest in the group VII leaves (**Figure 5C**). In comparison, the levels of nicotine in each group of leaves at each harvest date was higher in the second year than in the first year. The trends of benzenes were also similar in 2 years. The levels of benzenes were lower in each group of leaves at each harvest date in the first year than in the second year (**Supplementary Figure 6D**). The second type was that the levels of groups of metabolites in all or most samples groups (I–VII) were higher in the first year than in the second year. These groups of metabolites included triterpenes (**Figure 5D**), monoterpenes, diterpenes, amino acids, N-containing compounds (natural products), and polyphenols (**Supplementary Figures 6A, C, F, I**, and **J**). The accumulation patterns of triterpenes showed a trend from the highest in the group I leaves to the lowest in the group VII samples in 2 years (**Figure 5D**). Except for the group VI leaves, the levels of triterpenes in leaves were higher in the second year than in the first year. The levels of monoterpene, amino acids, and polyphenols in each group of samples at each harvest date were higher in the first year than in the second year. The third type trends for sesquiterpenes, PAH, organic acid, and sugar (**Supplementary Figures 6B, E, G, H**) ) were dynamic in the seven groups of samples between 2 years. Taken together, these results indicate that the metabolism of 12 groups of metabolites differentially respond to the growing years.

**Figure 6:**
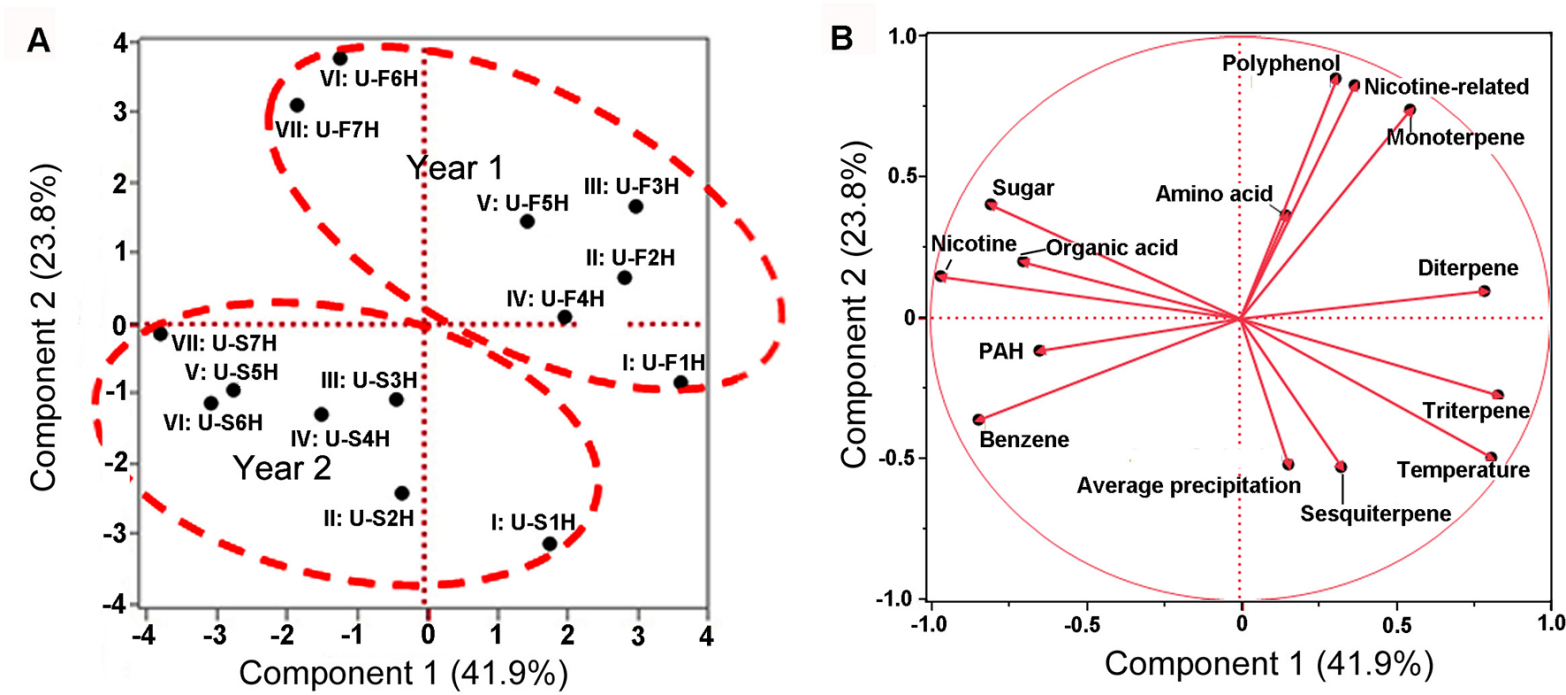
Scaling principal component analysis (PCA) showing associations between metabolite profiles with growing years and two climatic factors in North Carolina. This analysis was completed with JUMpro software. **(A)**, A plot of PCA shows an association between two growing years and metabolite profiles in seven groups of samples. In general, group samples showed a similar tendency in two consecutive years. **(B)**, A plot of PCA shows associations between two climatic factors and 12 groups of metabolites (including nicotine) as well as correlation among 12 groups of metabolites themselves in 2 years of field growth.

These 12 groups of metabolites, seven groups of samples, and 2 years were used as variables for PCA (with JUMpro software) to understand the potential correlation of growing years and metabolite profiles. The resulting two-dimensional ordination plot showed that the PC1 and the PC2 accounted for 41.9 and 23.8% of the total variance from 2 years in North Carolina (**Figure 6A**). In general, the seven group of samples were separated between the first and second years. The ordination plot also showed that in each year, the ordination order of seven group samples was relatively similar from I to VII. These data indicate that the positions (groups) of leaves and harvest times are associated with 12 groups of metabolite profiles.

These 12 groups of metabolites and two climatic factors (average temperature and precipitation values) were used as variables for PCA to understand ordination relevance. The resulting PCA plot showed an ordinate relevance between two climate factors and 12 groups of metabolites (**Figure 6B**). Temperature, precipitation, triterpene, and sesquiterpene fell in the same ordinate region, suggesting a close positive association. By contrast, nicotine, sugar, and organic acid fell in the opposite ordinate region against both temperature and precipitation, suggesting a negative ordinate relevance. Average precipitation also had a positive association with triterpene and sesquiterpene but had a negative relevance to nicotine, sugar, and organic acid. Other groups of metabolites were localized in two other ordinate regions, suggesting a potential partial relevance to these two weather factors.

Furthermore, a Pearson analysis was performed to evaluate the correlation between 12 groups of metabolites and these two climatic factors established by PCA. A bi-variate Pearson analysis revealed a linear relationship among variables (**Table 1**). The resulting data showed that temperature was positively associated with triterpene (P-value less than 0.01), sesquiterpene (P-value less than 0.05), and diterpene (P-value less than 0.05). By contrast, the resulting data showed that temperature was negatively associated with nicotine (P-value less than 0.01), sugar (P-value less than 0.01), and organic acid of TCA (P-value less than 0.05). The resulting data also showed a significantly positive association between average precipitation and triterpene (P-value less than 0.01).

**Table 1:** Data of Pearson correlation analysis for two weather facts and 12 groups of metabolites in North Carolina.

		Temperature	Daily precipitation	Monoterpene	Sesquiterpene	Diterpene	Triterpene	Benzene	PAH	Nicotine	Amino acid	Organic acid	Sugar	NCSM	Polyphenol
Temperature	Pearson correlation	1	0.225	0.057	0.556*	0.582*	0.767**	−0.519	−0.465	−0.855**	−0.119	−0.655*	−0.855**	−0.092	−0.176
	Sig. (two-tailed)		0.439	0.847	0.039	0.029	0.001	0.057	0.094	0	0.685	0.011	0	0.754	0.547
Daily precipitation	Pearson correlation	0.225	1	−0.328	0.194	0.08	0.553*	0.115	−0.106	−0.216	−0.032	0.16	−0.356	−0.309	−0.206
	Sig. (two-tailed)	0.439		0.252	0.507	0.786	0.040	0.695	0.719	0.458	0.914	0.584	0.212	0.283	0.480
Monoterpene	Pearson correlation	0.057	−0.328	1	−0.338	0.478	0.226	−0.747**	−0.33	−0.462	0.13	−0.376	−0.169	0.710**	0.819**
	Sig. (two-tailed)	0.847	0.252		0.237	0.084	0.436	0.002	0.249	0.096	0.657	0.186	0.565	0.004	0
Sesquiterpene	Pearson correlation	0.556*	0.194	−0.338	1	0.017	0.25	−0.009	−0.443	−0.357	0.099	−0.206	−0.511	−0.105	−0.285
	Sig. (two-tailed)	0.039	0.507	0.237		0.955	0.39	0.976	0.112	0.211	0.737	0.479	0.062	0.72	0.324
Diterpene	Pearson correlation	0.582*	0.08	0.478	0.017	1	0.722**	−0.604*	−0.222	−0.775**	0.267	−0.591*	−0.532	0.361	0.255
	Sig. (two-tailed)	0.029	0.786	0.084	0.955		0.004	0.022	0.446	0.001	0.356	0.026	0.05	0.205	0.379
Triterpene	Pearson correlation	0.767**	0.553*	0.226	0.25	0.722**	1	−0.571*	−0.388	−0.835**	0.104	−0.491	−0.752**	0.083	0.106
	Sig. (two-tailed)	0.001	0.04	0.436	0.39	0.004		0.033	0.171	0	0.722	0.074	0.002	0.777	0.717
Benzene	Pearson Correlation	−0.519	0.115	−0.747**	−0.009	−0.604*	−0.571*	1	0.718**	0.734**	−0.075	0.618*	0.452	−0.515	−0.543*
	Sig. (two-tailed)	0.057	0.695	0.002	0.976	0.022	0.033		0.004	0.003	0.799	0.019	0.105	0.06	0.045
PAH	Pearson correlation	−0.465	−0.106	−0.33	−0.443	−0.222	−0.388	0.718**	1	0.503	−0.237	0.274	0.45	−0.389	−0.314
	Sig. (two-tailed)	0.094	0.719	0.249	0.112	0.446	0.171	0.004		0.066	0.415	0.343	0.106	0.17	0.274
Nicotine	Pearson correlation	−0.855**	−0.216	−0.462	−0.357	−0.775**	−0.835**	0.734**	0.503	1	−0.01	0.747**	0.852**	−0.239	−0.179
	Sig. (two-tailed)	0	0.458	0.096	0.211	0.001	0	0.003	0.066		0.974	0.002	0	0.411	0.54
Amino acid	Pearson correlation	−0.119	−0.032	0.13	0.099	0.267	0.104	−0.075	−0.237	−0.01	1	0.262	−0.118	0.567*	0.242
	Sig. (two-tailed)	0.685	0.914	0.657	0.737	0.356	0.722	0.799	0.415	0.974		0.366	0.689	0.035	0.404
Organic acid	Pearson correlation	−0.655*	0.16	−0.376	−0.206	−0.591*	−0.491	0.618*	0.274	0.747**	0.262	1	0.53	0.097	0.088
	Sig. (two-tailed)	0.011	0.584	0.186	0.479	0.026	0.074	0.019	0.343	0.002	0.366		0.051	0.742	0.764
Sugar	Pearson correlation	−0.855**	−0.356	−0.169	−0.511	−0.532	−0.752**	0.452	0.45	0.852**	−0.118	0.53	1	−0.016	0.09
	Sig. (two-tailed)	0	0.212	0.565	0.062	0.05	0.002	0.105	0.106	0	0.689	0.051		0.956	0.76
NCSM	Pearson correlation	−0.092	−0.309	0.710**	−0.105	0.361	0.083	−0.515	−0.389	−0.239	0.567*	0.097	−0.016	1	0.844**
	Sig. (two-tailed)	0.754	0.283	0.004	0.72	0.205	0.777	0.06	0.17	0.411	0.035	0.742	0.956		0
Polyphenol	Pearson correlation	−0.176	−0.206	0.819**	−0.285	0.255	0.106	−0.543*	−0.314	−0.179	0.242	0.088	0.09	0.844**	1
	Sig. (two-tailed)	0.547	0.48	0	0.324	0.379	0.717	0.045	0.274	0.54	0.404	0.764	0.76	0	

Bold numbers mean significant difference. *significant correlation (2-tailed) with P-value less than 0.05, **significant correlation (2-tailed) with P-value less than 0.01, NCSM, nitrogen-containing secondary metabolites.

### Association of Two Climatic Factors and 12 Groups of Metabolites in India

**Figure 7:**
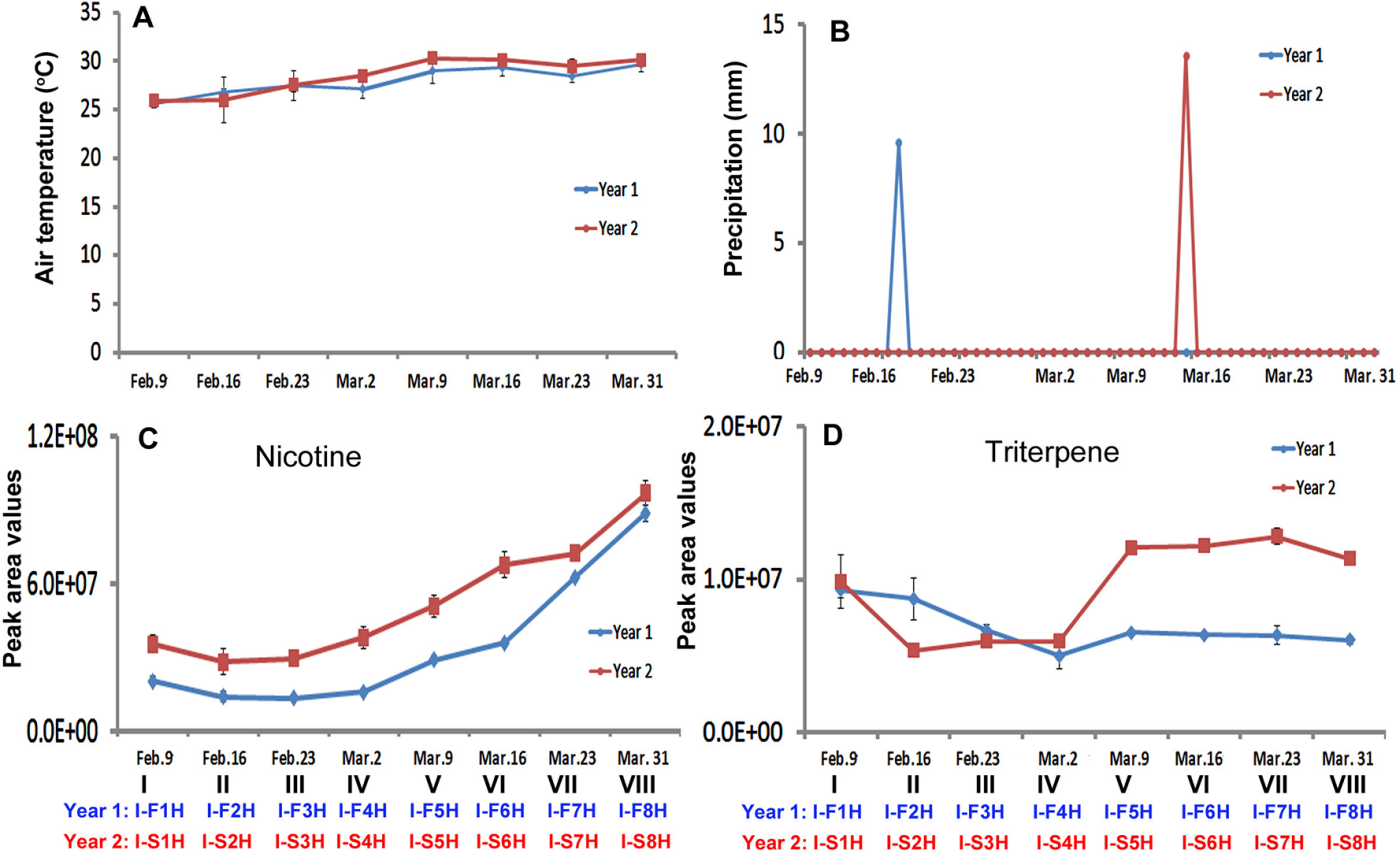
Dynamics of two climate factors and trends of two groups of metabolites in two harvesting seasons in India. **(A–B)**, Dynamics of average temperature **(A)** and precipitation **(B)** values at eight harvesting dates. Each value resulted from averaging daily temperature or precipitation levels in that week of harvest. **(C–D)** Trends of total nicotine levels **(C)** triterpene levels **(D)** in eight groups of samples harvested on the eight harvesting dates.

As described above, daily temperature and precipitation values were recorded at the field in the two growing seasons in India. The average temperature values were calculated for each harvest week. During the entire growing seasons, the average temperature values showed an increased trend from Feb. 9 through March 31 in both years (**Figure 7A**). In the week of Feb. 9, Feb. 23, and Mar. 31, average temperature values were very close in 2 years, while the temperature values in other weeks of March were higher in the second year than in the first year. Regarding daily precipitation, it was mostly dry during the harvest period in the two growing years (**Figure 7B**). From Feb. 9 to March 31, it rained only once in each year, on Feb. 20 in the first year and on March 13 in the second year (**Figure 7B**).

The trends of nicotine and 11 groups of metabolites were analyzed with their total peak values in eight groups (different positional) of leaves collected on eight dates from Feb. 9 through Mar. 31. The resulting plots showed three types of dynamic trends. The first trend was that the levels of four groups of metabolites in all groups (at all dates) or groups V–VIII (at the late half dates) were higher in the second year than in the first year. Nicotine, triterpenes, benzenes, and PAH followed this trend (**Figures 7C, D** and **Supplementary Figures 7D** and **E**). The nicotine levels in the eight groups of leaves showed an increased trend in two years (**Figure 7C**). In comparison, the levels of nicotine in each group of leaves were higher in the second year than in the first year. The second trend was that the total levels of four groups of metabolites in all groups (all dates) or most groups (most dates) samples were higher in the first year than in the second year. These groups of metabolites included monoterpenes, sesquiterpenes, diterpenes, and polyphenols (**Supplementary Figures 7A–C, J** ). The third trend was that the levels of four groups of metabolites were dynamic in eight groups of samples in the 2 years. These includes, amino acids, organic acids, and sugars (**Supplementary Figures 7F, G, H, and I**). Although the levels of sugars were dynamic, their values increased from I-F1H and I-S1H through I-F8H and I-S8H (**Supplementary Figure 7H**).

**Figure 8:**
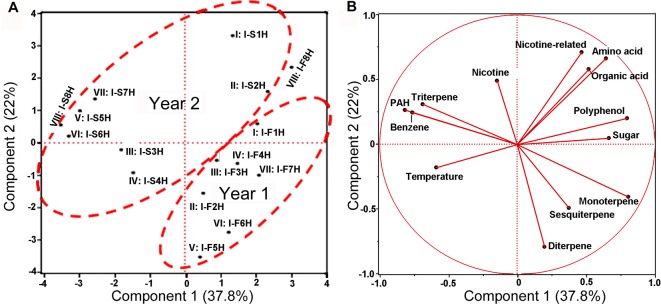
Scaling principal component analysis (PCA) showing associations between metabolite profiles with growing years and two climatic factors in India. This analysis was completed with JUMpro software. **(A)**, A plot of PCA results show an association between two growing years and metabolite profiles in eight groups of samples. **(B)**, A plot of PCA results shows associations between 2-year’s temperature and 12 groups of metabolites (including nicotine) as well as correlations among 12 metabolites themselves in 2 years of field growth.

PCA (Eigen-vector based scaling) was performed to characterize the correlation of 12 groups of metabolites, 2 years, and eight groups of samples. The resulting plot showed that the PC1 and the PC2 accounted for 35.2 and 22.7% of the total variance from two different years (**Figure 8A**). Eight groups of samples (I–VIII) in the first and second years could be ordinately grouped together, respectively. In comparison, the ordinate association was better in the first year than in the second year. These results indicate differential effects of the two growing years on the profiles of 12 group metabolites.

Eight average temperature values, eight average precipitation values, and 12 groups of metabolites were used as variables for PCA. The resulting PCA plot showed potential ordinate relevance between temperature and 12 groups of metabolites (**Figure 8B**). Given that most of precipitation values were zero, precipitation was absent in the plot. This plot showed an opposite ordinate relevance between temperature and sugars, polyphenol, amino acids, organic acids of TCA, and nitrogen-containing secondary metabolites, suggesting a potential negative association. By contrast, this plot showed a close ordinate relevance of temperature with PAH, triterpenes, and benzene in the PC1 axis, suggesting a potential positive association. Pearson analysis was carried out using eight average temperature values, levels of 12 groups of metabolites in 2 years, and eight groups of samples. As described in PCA, precipitation was not included due to almost no rain. A bi-variate Pearson analysis revealed a linear relationship among variables (**Table 2**). The resulting correlation parameters showed that temperature was positively correlated with benzene and PAH (P-values less than 0.05), but negatively correlated with amino acid and organic acid (P-values less than 0.05).

**Table 2:** Data of Pearson correlation analysis for temperature and 12 groups of metabolites in India.

		Temperature	Monoterpene	Sesquiterpene	Diterpene	Triterpene	Benzene	PAH	Nicotine	Amino acid	Organic acid	Sugar	NCSM	Polyphenol
Temperature	Pearson correlation	1	−0.3	0.186	−0.1	0.38	0.613*	0.499*	0.12	−0.551*	−0.588*	−0.024	−0.207	−0.236
Sig. (two-tailed)		0.26	0.49	0.714	0.147	0.012	0.049	0.659	0.027	0.017	0.931	0.442	0.379	
Monoterpene	Pearson correlation	−0.3	1	0.559*	0.488	−0.477	−0.580*	−0.642**	−.523*	0.317	0.145	0.633**	0.158	0.672**
Sig. (two-tailed)	0.26		0.024	0.055	0.062	0.018	0.007	0.038	0.231	0.591	0.009	0.56	0.004	
Sesquiterpene	Pearson correlation	0.186	0.559*	1	0.528*	−0.23	−0.245	−0.203	−0.152	−0.05	−0.155	0.581*	0.108	0.211
Sig. (two-tailed)	0.49	0.024		0.035	0.391	0.361	0.451	0.575	0.856	0.567	0.018	0.691	0.432	
Diterpene	Pearson correlation	−0.1	0.488	0.528*	1	−0.309	−0.305	−0.313	−0.425	−0.317	−0.078	−0.063	−0.371	−0.222
Sig. (two-tailed)	0.714	0.055	0.035		0.244	0.251	0.238	0.101	0.232	0.773	0.818	0.158	0.41	
Triterpene	Pearson correlation	0.38	−0.477	−0.23	−0.309	1	0.802**	0.811**	−0.079	−0.068	−0.138	−0.251	−0.067	−0.454
Sig. (two-tailed)	0.147	0.062	0.391	0.244		0	0	0.772	0.803	0.61	0.348	0.805	0.077	
Benzene	Pearson correlation	0.613*	−0.580*	−0.245	−0.305	0.802**	1	0.803**	−0.046	−0.199	−0.207	−0.232	−0.167	−0.422
Sig. (two-tailed)	0.012	0.018	0.361	0.251	0		0	0.867	0.46	0.443	0.386	0.536	0.103	
PAH	Pearson correlation	0.499*	−0.642**	−0.203	−0.313	0.811**	0.803**	1	0.131	−0.292	−0.317	−0.351	−0.034	−0.575*
Sig. (two-tailed)	0.049	0.007	0.451	0.238	0	0		0.627	0.273	0.231	0.182	0.902	0.02	
Nicotine	Pearson correlation	0.12	−0.523*	−0.152	−0.425	−0.079	−0.046	0.131	1	−0.021	0.087	−0.151	0.425	−0.062
Sig. (two-tailed)	0.659	0.038	0.575	0.101	0.772	0.867	0.627		0.939	0.749	0.578	0.101	0.821	
Amino acid	Pearson correlation	−0.551*	0.317	−0.05	−0.317	−0.068	−0.199	−0.292	−0.021	1	0.805**	0.504*	0.759**	0.595*
Sig. (two-tailed)	0.027	0.231	0.856	0.232	0.803	0.46	0.273	0.939		0	0.046	0.001	0.015	
Organic acid	Pearson correlation	−0.588*	0.145	−0.155	−0.078	−0.138	−0.207	−0.317	0.087	0.805**	1	0.149	0.603*	0.315
Sig. (two-tailed)	0.017	0.591	0.567	0.773	0.61	0.443	0.231	0.749	0		0.582	0.013	0.235	
Sugar	Pearson correlation	−0.024	0.633**	0.581*	−0.063	−0.251	−0.232	−0.351	−0.151	0.504*	0.149	1	0.426	0.780**
Sig. (two-tailed)	0.931	0.009	0.018	0.818	0.348	0.386	0.182	0.578	0.046	0.582		0.1	0	
NCSM	Pearson correlation	−0.207	0.158	0.108	−0.371	−0.067	−0.167	−0.034	0.425	0.759**	0.603*	0.426	1	0.49
Sig. (two-tailed)	0.442	0.56	0.691	0.158	0.805	0.536	0.902	0.101	0.001	0.013	0.1		0.054	
Polyphenol	Pearson correlation	−0.236	0.672**	0.211	−0.222	−0.454	−0.422	−.575*	−0.062	0.595*	0.315	0.780**	0.49	1
Sig. (two-tailed)	0.379	0.004	0.432	0.41	0.077	0.103	0.02	0.821	0.015	0.235	0	0.054		

Bold numbers mean significant difference. *significant correlation (2-tailed) with P-value less than 0.05, **significant correlation (2-tailed) with P-value less than 0.01, NCSM, nitrogen-containing secondary metabolites.

### Leaf Nicotine Trends Are Not Altered by Two Growing Locations

Although our experimental design did not specifically target nicotine, we used untargeted data to compare nicotine profiles in leaves from India *vs*. North Carolina. The rationale is that unlike other metabolites, nicotine is the signature metabolite of tobacco, which is biosynthesized in roots and stored in leaves. The resulting data showed that although the levels of nicotine between 2 years were altered in samples from both India and North Carolina, the profile trends from the bottom to top leaves were similar (**Figures 5C**, **7C**). Pearson analysis showed a negative association between nicotine profiles and temperature in North Carolina in 2 years. By contrast, Pearson analysis did not show a significant association between temperature and nicotine profiles in India. In addition, there was not a significant association between precipitation and nicotine profiles in North Carolina. Pearson analysis could not be preformed to evaluate the association between precipitation and nicotine profiles in India due to almost no rain in two farming seasons (**Figure 7B**). In summary, these findings show that the trends of nicotine levels from the bottom to top leaves are independent upon local climates and farming locations.

## Discussion

### Association of Growing Locations and Plant Metabolomes

To date, environmental changes have been reported to highly influence the sensitivity of plants to environments and plant productivity (Aggarwal, 2008; Ahmad et al., 2010; Walter et al., 2010; Gosling et al., 2011). Furthermore, numerous studies have particularly reported that global climate change can highly affect agricultural productivity and crop yield (Walter et al., 2010; Gosling et al., 2011; Bowen and Friel, 2012; Dwivedi et al., 2013). Most of this type of research has focused on single climate factors, such as temperature in a controlled condition or a specific local environment. A recent study was completed to understand the metabolic responses of rice to temperature changes (Glaubitz et al., 2015). This study used 12 rice cultivars, having different sensitivity to high temperature conditions, to understand their metabolic reactions in vegetative tissues in a given condition. The results showed that the tricarboxylic acid cycle and amino acid biosynthesis in sensitive cultivars were significantly affected by high night temperature and the levels of certain metabolites such as putrescine, spermidine, and spermine were increased in sensitive cultivars (Glaubitz et al., 2015). Another greenhouse study showed that effects of elevated CO_2_, elevated temperature, and water deficit alone or in combination differentially affected grape performance in both grape yield and plant growth in a cultivar-dependent manner (Kizildeniz et al., 2015). Drought under an elevated temperature was found to drastically inhibit the vegetative growth withreduced bunch fresh and dry weights of two grape cultivars. Increased carbon dioxide, elevated temperature, and drought were found to reduce the total polyphenolics (Kizildeniz et al., 2015). Similar studies have been completed to understand the effects of single or combined climate factors on other crop growth and production (Sengar et al., 2015). However, studies on plant metabolomes responsive to global climate changes are limited. Moreover, effects of real field climate on crop metabolism in the farming field remains largely open for investigation. In the study reported herein, the objective was to use the globally commercialized K326 tobacco cultivar to understand the effects of real field environment conditions on plant metabolome. We selected two research station fields in two completely different environmental conditions, India and USA. The planting of K326 and field management were exactly followed as per location-specific farming protocols. Using an untargeted metabolomics to profile metabolites we were able toannotate 171 non-polar metabolites from 387 assays and 225 polar metabolites from 366 assays. Further PCA and HCA using these metabolites provided the same result that although plants were grown in two continents, the non-polar and polar metabolomes annotated were primarily grouped and clustered by growing years (**Figures 1**, **2**, **3**, and **4**). These results indicated that the two growing years played a primary role leading to differentiation of metabolomes. Moreover, plants were grown in the same place and managed with the same field protocols during both the years. Both Pearson analysis and PCA revealed positive or negative relevance between temperature and six groups of metabolites in two years in North Carolina (**Table 1**). In addition, precipitation in North Carolina was also significantly associated with triterpenes (**Table 1**). These results suggest that climate changes between 2 years are the main factor associated with the observed plant metabolism differentiation.

### Effects of Field Air Temperature on Metabolome

All environmental factors, such as temperature, precipitation, intensity of light, photoperiod, soil condition, wind, pests, diseases, ultraviolet lights, nutrients, soil moisture, and others can significantly affect plant growth and metabolism in the field. Each environmental factor plays a significant role. Therefore, metabolite profile data obtained from 2 years of investigation actually resulted from the comprehensive effect of all field factors in two growing locations. We understood that data for all environmental factors were essential to understand their effects on tobacco metabolism. Although it was difficult to track accurate data of all factors, we could obtain accurate values (hourly and daily) for two weather factors from local weather stations, air temperature and precipitation. Therefore, both air temperature and precipitation of two growing seasons were used to understand their association with metabolite profiles.

Temperature has been reported a primary factor that can affect plant metabolism. Single temperature factor in the control condition has been showed to affect plant metabolism in different ways. A genome-wide study of *Arabidopsis* indicated metabolic network changes caused by different temperature conditions (Topfer et al., 2013). The increase of certain amino acids during seed imbibition of *Ricinus communis* was reported to be responsive to temperature increase to 35°C (Ribeiro et al., 2015). Furthermore, the reprogramming of the metabolome was reported to occur in temperature stress. For example, the central carbohydrate metabolism can be regulated by the temperature-stress (Guy et al., 2008). In our study, we used Pearson analysis to characterize association of the field air temperatures in two different geographical areas with leaf metabolome composition. To understand the potential association of temperature and metabolome, we selected nicotine and 11 groups of metabolites (**Figures 5** and **7**, **Supplementary Figures 6** and **7**, and **Supplementary Table 9**). The 11 groups included both plant primary and secondary metabolites (**Supplementary Table 9**). The total level of each group at different harvest date was summed from individual metabolites, and then used for characterization of their level trends, PCA, and Pearson analysis. The resulting data allowed evaluating association of metabolite groups with field air temperature. We selected nicotine as a representative metabolite, because this health-associated alkaloid is the signature metabolite of tobacco. The resulting dynamic trend data developed from each harvest time, PCA, and Pearson analysis simultaneously showed that the levels of nicotine were negatively associated with field air temperatures in 2 years of growth in North Carolina (**Figures 5A, C**, **6B**, **Supplementary Figure 6D**, and **Table 1**). By contrast, although the trends of temperature and nicotine levels in India (**Figures 7A, C**) were similar to those in North Carolina, the results from PCA and Pearson analysis showed a potentially partial association (**Figure 8B** and **Table 2**). This observation was consistent with our previous data reporting nicotine differentiation in tobacco samples from India, USA, and Brazil (Ma et al., 2013).

In addition, we observed the associations between temperature and other metabolites. It was interesting to note that the total level of organic acids of TCA cycle (**Supplementary Table 8**) was negatively associated with temperature in both North Carolina and India (**Tables 1** and **2**). One of reduced organic acids is malonic acid, an intermediate in the TCA cycle (Fernie et al., 2004). The reduction of this acid and other acids of TCA by high temperature was also observed in soybean (Sicher, 2013). These data suggest that the accumulation of organic acids of TCA is associated with the field air temperature. Moreover, we observed the different responses of metabolites to temperatures in two locations. In North Carolina, temperature was positively associated with sesquiterpenes, diterpenes, and triterpenes, but negatively associated with sugars (**Table 1**). In India, temperature was positively associated with PAH and benzene but was negatively associated with amino acids. These responsive differences likely result from multiple environmental factors in the field. Experimental evidence has showed that a combination of environmental factors such as heat shock and drought can lead to multiple alterations in plant metabolism, as in photosynthesis and enzyme activity (Rizhsky et al., 2002). Extreme temperature (together with water deficit) and high solar radiation were reported to strongly affect grapevine growth, which further led to negative effects on fruit and wine quality (Teixeira et al., 2014). A field study in China revealed that different field conditions also affected the profiles of polar metabolites in tobacco leaves such as flavonoids (Li et al., 2014). In 2 years of our field experiments, it rained only once during each year in India. We hypothesize that this extreme precipitation event can affect effects of temperature on plant metabolism in India. In summary, temperature conditions play a primary role in regulating plant metabolism in the field. We further believe that as more field experiments will be performed, continuous documentation of data will enhance the understanding of combined effects of temperature and other factors on plant metabolism.

### Effects of Precipitation on Metabolome

Global changes in precipitation regimes have been continuously documented (Keller, 2007; Schnoor, 2007). Those precipitation changes are associated with different factors, such as rising temperature (Le Roux et al., 2013; Vlam et al., 2014). Variation in precipitation patterns such as water deficit (drought) have been reported to lead to lower the photosynthetic rates and plant growth as well as other physiological features (Munne-Bosch and Penuelas, 2003; Tezara et al., 2003). In comparison, studies on effects of variation in precipitation patterns such as drought on metabolites accumulation remain open for study. In this study, we recorded daily precipitation and calculated average precipitation during the growing seasons in two continuous years. The weather was dry in India during these 2 years, whereinthe field received raining only onceduring the growing season in each year (**Figure 7B**). Consequently, we could only analyze relevance of precipitation and metabolomes for North Carolina samples. The weekly average and daily precipitation values were used for PCA and Pearson analysis, respectively. The resulting PCA data showed a positive relevance among average precipitation, temperature, triterpenes, and sesquiterpenes (**Figure 6B**). The resulting Pearson analysis data showed a significant association between daily precipitation and triterpenes. These data provide evidence that precipitation can affect plant metabolism, although it is hard to predict precipitation year by year.

### Leaf Nicotine Trends Independent of Environmental Factors

Nicotine is the main signature metabolite of tobacco. It is formed in the roots, transported to above ground tissues, and stored in the leaves. Our study reveals an interesting metabolic independence of nicotine level trends upon climate from 2 years of studies. This independent feature is that although Pearson analysis and PCA showed positive and negative associations between nicotine and temperature in North Carolina and India, respectively, the trend of nicotine levels was not altered in 2 years of experiments. In two fields, the bottom leaves had lower nicotine levels, while the top leaves had the higher nicotine levels (**Figures 6C** and **7C**). In contrast, the level trends of 11 other groups of metabolites in different groups of samples were dynamic in tobacco leaves from both North Carolina and India. These interesting results indicate that the trend of nicotine levels is stably controlled by tobacco plants. The mechanism behind this trend stability of nicotine remain uninvestigated, although its biosynthetic pathway has been studied intensively(Xie and Fan, 2016; Kajikawa et al., 2017). Furthermore, it has been vastly documented that nicotine is exclusively biosynthesized in roots and then transported to leaves for storage. In our study, we used standardized tobacco farming protocols to grow plants in the field. Therefore, the use of fertilizer and the time of fertilization were consistent in 2 years. Based on these observationswe hypothesize that although temperature and other environmental factors can affect nicotine levels in leaves, weather factors might have limited effects on the trend of nicotine levels controlled by the root-specific biosynthesis. This finding shows that it is interesting to understand effects of environmental factors on the formation of root-specific metabolites in the future.

## Conclusion Remarks

Herein, we integrate unbiased un-targeted metabolomics with standard farming practices followed for commercial cultivation oftobacco to understand the formation of root-specific effects of field environments on plant metabolism. From a large number of peaks detected, we could annotate 171 non-polar and 225 polar metabolites and further grouped them into different classes. Nicotine, the main tobacco alkaloid, is one of the non-polar metabolites and used as signature metabolite in our data analysis. PCA and HCA characterized that two different years and two different geographical locations played primary and secondary roles in controlling metabolite complexity. Field air temperature was characterized to be one of the main factors that was associated with profiles of several main groups of metabolites. We further used nicotine as a signature metabolite to indicate that its profile trend in different leaves controlled by root-specific biosynthesis is independent of climate factors. This pilot study provides useful findings to enhance the understanding of the effects of two diverse climate factors on plant metabolism across two continents.

## Data Availability Statement

The raw data supporting the conclusions of this manuscript will be made available by the authors, without undue reservation, to any qualified researcher.

## Author Contributions

D-YX and SG conceived this research and designed all experiments in North Carolina and India, respectively. D-YX and SG also participated in sample collection and collected weather data in North Carolina and India, respectively. D-YX participated in data analysis and figure preparation, and drafted and finalized this manuscript. D-MM managed plant growth in field in North Carolina, collected samples, performed metabolic extraction and GC-MS analysis and other lab experiments, analyzed data, and prepared figures and manuscript. CH participated in field design and management of plant growth, involved in sampling, and metabolite extraction. RM participated in field design and management and sampling in India.

## Conflict of Interest

The authors declare that the research was conducted without any commercial or financial relationships that could be construed as a potential conflict of interest.
