# Supplementary material for: Untargeted Metabolomics of Nicotiana tabacum Grown in United States and India Characterizes the Association of Plant Metabolomes With Natural Climate and Geography
Source: Front Plant Sci. 2019 Oct 30;10:1370. doi: 10.3389/fpls.2019.01370 (PMC6831618; doi:10.3389/fpls.2019.01370)
Supplement: Supplementary file 8 [file Table_1.docx]

Supplementary Table 1 Sample harvesting dates and labeling for metabolomics of leaves in North Carolina

| **Harvest time** | **Date** | **Sample labeling in the field** | **Leaf number and group labelling used in PCA and HCA** |
| --- | --- | --- | --- |
| First harvest (leaves 1-2) | Aug. 3^rd^ | MP1R1- I (1-10 plants in plot 1)  MP1R2- I (11-20 plants in plot 1)  MP1R3- I (61-70 plants in plot 2)  MP1R4- I (71-80 plants in plot 2)  MP1R5- I (121-130 plants in plot 3)  MP1R6- I (131-140 plants in plot 3) | I: 1-2 leaves  **U-F1H** for the first year, 6 biological samples  **U-S1H** for the second year, 6 biological samples |
| Second harvest (leaves 3-8) | Aug. 24^th^ | MP2R1- II, III (1-10 plants in plot 1)  MP2R2- II, III (11-20 plants in plot 1)  MP2R3- II , III (61-70 plants in plot 2)  MP2R4- II, III (71-80 plants in plot 2)  MP2R5- II, III (121-130 plants in plot 3)  MP2R6- II, III (131-140 plants in plot 3) | II: 3-5 leaves  **U-F2H** for the first year, 6 biological samples  **U-S2H** for the second year, 6 biological samples  III: 6-8 leaves  **U-F3H** for the first year, 6 biological samples  **U-S3H** for the second year, 6 biological samples |
| Third harvest (leaves 9-14) | Sept. 14^th^ | MP3R1- IV, V (1-10 plants in plot 1)  MP3R2- IV, V (11-20 plants in plot 1)  MP3R3- IV, V (61-70 plants in plot 2)  MP3R4- IV, V (71-80 plants in plot 2)  MP3R5- IV, V (121-130 plants in plot 3)  MP3R6- IV, V (131-140 plants in plot 3) | IV: 9-11 leaves  **U-F4H** for the first year, 6 biological samples  **U-S4H** for the second year, 6 biological samples  V: 12-14 leaves  **U-F5H** for the first year, 6 biological samples  **U-S5H** for the second year, 6 biological samples |
| Forth harvest (leaves 15-20) | Oct. 4^th^ | MP4R1- VI, VII (1-10 plants in plot 1)  MP4R2- VI, VII (11-20 plants in plot 1)  MP4R3- VI, VII (61-70 plants in plot 2)  MP4R4- VI, VII (71-80 plants in plot 2)  MP4R5- VI, VII (121-130 plants in plot 3)  MP4R6- VI, VII (131-140 plants in plot 3) | VI: 15-17 leaves;  **U-F6H** for the first year, 6 biological samples  **U-S6H** for the second year, 6 biological samples  VII: 18-20 leaves  **U-F7H** for the first year, 6 biological samples  **U-S7H** for the second year, 6 biological samples |

Each leaf will be cut into two sections. One including midrib for curing and the other half frozen in liquid nitrogen and stored in freezer. MP1R1-I: Metabolomics Pick #1 Replicate 1- I group. PCA and HCA: principal component analysis and hierarchical clustering analysis. Groups of U-F1H through U-F7H were USA’s samples in the First year from positions 1 to 7 Harvest. Groups of U-S1H through U-S7H were USA’s samples in the Second year from positions 1 to 7 Harvest.
