# Supplementary material for: Untargeted Metabolomics of Nicotiana tabacum Grown in United States and India Characterizes the Association of Plant Metabolomes With Natural Climate and Geography
Source: Front Plant Sci. 2019 Oct 30;10:1370. doi: 10.3389/fpls.2019.01370 (PMC6831618; doi:10.3389/fpls.2019.01370)
Supplement: Supplementary file 9 [file Table_2.docx]

Supplementary Table 2 Control sampling (entire leaf) harvesting dates and labeling for metabolomics of leaves in North Carolina

| **Harvest time** | **Date** | **Sample labeling** | **Leaf number and grouping** |
| --- | --- | --- | --- |
| First harvest (leaves 1-2) | Aug. 3^rd^ | CMP1R1-I (21-25 plants in plot 1)  CMP1R2-I (81-85 plants in plot 2)  CMP1R3-I (141-145 plants in plot 3) | I: 1-2 leaves  (3 biological samples each year) |
| Second harvest (leaves 3-8) | Aug. 24^th^ | CMP2R1-II, III (21-25 plants in plot 1)  CMP2R2-II, III (81-85 plants in plot 2)  CMP2R3-II, III (141-145 plants in plot 3) | II: 3-5 leaves  III: 6-8 leaves  (6 biological samples each year) |
| Third harvest (leaves 9-14) | Sept. 14^th^ | CMP3R1-IV, V (21-25 plants in plot 1)  CMP3R2-IV, V (81-85 plants in plot 2)  CMP3R3-IV, V (141-145 plants in plot 3) | IV: 9-11 leaves  V: 12-14 leaves  (6 biological samples, each year) |
| Forth harvest (leaves 15-20) | Oct. 4^th^ | CMP4R1-VI, VII (21-25 plants in plot 1)  CMP4R2-VI, VII (81-85 plants in plot 2)  CMP4R3-VI, VII (141-145 plants in plot 3) | VI: 15-17 leaves  VII: 18-20 leaves  (6 biological samples each year) |

CMP1R1-I: control metabolomics pick #1 replicate-1-I group.
