# Supplementary material for: Untargeted Metabolomics of Nicotiana tabacum Grown in United States and India Characterizes the Association of Plant Metabolomes With Natural Climate and Geography
Source: Front Plant Sci. 2019 Oct 30;10:1370. doi: 10.3389/fpls.2019.01370 (PMC6831618; doi:10.3389/fpls.2019.01370)
Supplement: Supplementary file 10 [file Table_3.docx]

Supplementary Table 3 Sampling dates and labeling for metabolomics of samples from India

| **Harvest time** | **Date** | **Sample labeling in the field** | **Leaf number and groups used in PCA and HCA** |
| --- | --- | --- | --- |
| First harvest (leaves 1-2) | Feb. 9^th^ | MP1R1- I (1-10 plants in plot 1)  MP1R2- I (11-20 plants in plot 1)  MP1R3- I (61-70 plants in plot 2)  MP1R4- I (71-80 plants in plot 2)  MP1R5- I (121-130 plants in plot 3)  MP1R6- I (131-140 plants in plot 3) | I: 1-2 leaves  **I-F1H** for the first year, 6 biological samples  **I-S1H** for the second year , 6 biological samples |
| Second harvest (leaves 3-5) | Feb. 16^th^ | MP2R1- II (1-10 plants in plot 1)  MP2R2- II (11-20 plants in plot 1)  MP2R3- II (61-70 plants in plot 2)  MP2R4- II (71-80 plants in plot 2)  MP2R5- II (121-130 plants in plot 3)  MP2R6- II (131-140 plants in plot 3) | II: 3-5 leaves  **I-F2H** for the first year, 6 biological samples  **I-S2H** for the second year, 6 biological samples |
| Third harvest (leaves 6-8) | Feb. 23^rd^ | MP3R1- III (1-10 plants in plot 1)  MP3R2- III (11-20 plants in plot 1)  MP3R3- III (61-70 plants in plot 2)  MP3R4- III (71-80 plants in plot 2)  MP3R5- III (121-130 plants in plot 3)  MP3R6- III (131-140 plants in plot 3) | III: 6-8 leaves  **I-F3H** for the first year, 6 biological samples  **I-S3H** for the second year, 6 biological samples |
| Forth harvest (leaves 9-11) | March 2^nd^ | MP4R1- IV (1-10 plants in plot 1)  MP4R2- IV (11-20 plants in plot 1)  MP4R3- IV (61-70 plants in plot 2)  MP4R4- IV (71-80 plants in plot 2)  MP4R5- IV (121-130 plants in plot 3)  MP4R6- IV (131-140 plants in plot 3) | IV: 9-11 leaves  **I-F4H** for the first year, 6 biological samples  **I-S4H** for the second year, 6 biological samples |
| Fifth harvest (leaves 12-14) | March. 9th | MP5R1- I (1-10 plants in plot 1)  MP5R2- I (11-20 plants in plot 1)  MP5R3- I (61-70 plants in plot 2)  MP5R4- I (71-80 plants in plot 2)  MP5R5- I (121-130 plants in plot 3)  MP5R6- I (131-140 plants in plot 3) | V: 12-14 leaves  **I-F5H** for the first year, 6 biological samples  **I-S5H** for the second year, 6 biological samples |
| Sixth harvest (leaves 15-17) | March. 16th | MP6R1- VI (1-10 plants in plot 1)  MP6R2- VI (11-20 plants in plot 1)  MP6R3- VI (61-70 plants in plot 2)  MP6R4- VI (71-80 plants in plot 2)  MP6R5- VI (121-130 plants in plot 3)  MP6R6- VI (131-140 plants in plot 3) | VI: 15-17 leaves  **I-F6H** for the first year, 6 biological samples  **I-S6H** for the second year, 6 biological samples |
| Seventh harvest (leaves 18-20) | March. 23rd | MP7R1- VII (1-10 plants in plot 1)  MP7R2- VII (11-20 plants in plot 1)  MP7R3- VII (61-70 plants in plot 2)  MP7R4- VII (71-80 plants in plot 2)  MP7R5- VII (121-130 plants in plot 3)  MP7R6- VII (131-140 plants in plot 3) | VII: 18-20 leaves  **I-F7H** for the first year, 6 biological samples  **I-S7H** for the second year, 6 biological samples |
| Eighth harvest (leaves 21-22) | March. 31st | MP8R1- VIII (1-10 plants in plot 1)  MP8R2- VIII (11-20 plants in plot 1)  MP8R3- VIII (61-70 plants in plot 2)  MP8R4- VIII (71-80 plants in plot 2)  MP8R5- VIII (121-130 plants in plot 3)  MP8R6- VIII (131-140 plants in plot 3) | VIII: 21-22 leaves  **I-F8H** for the first year, 6 biological samples  **I-S8H** for the second year, 6 biological samples |

Each leaf was cut into two sections. One including midrib for curing and the other half frozen in liquid nitrogen and stored in freezer. PCA and HCA: principal component analysis and hierarchical clustering analysis. MP1R1-I: Metabolomics Pick #1 Replicate 1- I group. Groups I-F1H through I-F8H were India’s samples in the First year from positions 1 to 8 Harvest. Groups of I-S1H through I-S8H were India’s samples in the Second year from positions 1 to 8 Harvest.
