# Supplementary material for: Untargeted Metabolomics of Nicotiana tabacum Grown in United States and India Characterizes the Association of Plant Metabolomes With Natural Climate and Geography
Source: Front Plant Sci. 2019 Oct 30;10:1370. doi: 10.3389/fpls.2019.01370 (PMC6831618; doi:10.3389/fpls.2019.01370)
Supplement: Supplementary file 11 [file Table_4.docx]

Supplementary Table 4 Control (entire leaf) sampling dates and labeling for metabolomics of leaves in India

| **Harvest time** | **Date** | **Sample labeling** | **Leaf number and grouping** |
| --- | --- | --- | --- |
| First harvest (leaves 1-2) | Feb. 9^th^ | CMP1R1- I (1-10 plants in plot 1)  CMP1R2- I (11-20 plants in plot 1)  CMP1R3- I (61-70 plants in plot 2) | I: 1-2 leaves  3 biological samples each year |
| Second harvest (leaves 3-5) | Feb. 16^th^ | CMP2R1- II (1-10 plants in plot 1)  CMP2R2- II (11-20 plants in plot 1)  CMP2R3- II (61-70 plants in plot 2) | II: 3-5 leaves  3 biological samples each year |
| Third harvest (leaves 6-8) | Feb. 23^rd^ | CMP3R1- III (1-10 plants in plot 1)  CMP3R2- III (11-20 plants in plot 1)  CMP3R3- III (61-70 plants in plot 2) | III: 6-8 leaves  3 biological samples each year |
| Forth harvest (leaves 9-11) | March 2^nd^ | CMP4R1- IV (1-10 plants in plot 1)  CMP4R2- IV (11-20 plants in plot 1)  CMP4R3- IV (61-70 plants in plot 2) | IV: 9-11 leaves  3 biological samples each year |
| Fifth harvest (leaves 12-14) | March. 9^th^ | CMP5R1- I (1-10 plants in plot 1)  CMP5R2- I (11-20 plants in plot 1)  CMP5R3- I (61-70 plants in plot 2) | V: 12-14 leaves  3 biological samples each year |
| Sixth harvest (leaves 15-17) | March. 16^th^ | CMP6R1- VI (1-10 plants in plot 1)  CMP6R2- VI (11-20 plants in plot 1)  CMP6R3- VI (61-70 plants in plot 2) | VI: 15-17 leaves  3 biological samples each year |
| Seventh harvest (leaves 18-20) | March. 23^rd^ | CMP7R1- VII (1-10 plants in plot 1)  CMP7R2- VII (11-20 plants in plot 1)  CMP7R3- VII (61-70 plants in plot 2) | VII: 18-20 leaves  3 biological samples each year |
| Eighth harvest (leaves 21-22) | March. 31^st^ | CMP8R1- VIII (1-10 plants in plot 1)  CMP8R2- VIII (11-20 plants in plot 1)  CMP8R3- VIII (61-70 plants in plot 2) | VIII: 21-22 leaves  3 biological samples each year |

CMP1R1-I: control metabolomics pick #1 replicate-1-I group.
