# Supplementary material for: Untargeted Metabolomics of Nicotiana tabacum Grown in United States and India Characterizes the Association of Plant Metabolomes With Natural Climate and Geography
Source: Front Plant Sci. 2019 Oct 30;10:1370. doi: 10.3389/fpls.2019.01370 (PMC6831618; doi:10.3389/fpls.2019.01370)
Supplement: Supplementary file 13 [file Table_6.docx]

Supplementary Table 6 one hundred and seventy-one non-polar metabolites annotated from 387 samples

| Categories | Metabolites Annotated |
| --- | --- |
| Hydrocarbons | 1,19-Eicosadiene [ C20H38, CAS ID=14811-95-1 ] |
|  | 10-Methylnonadecane [ C20H42, CAS ID=56862-62-5 ] |
|  | 10-Methyl-octadec-1-ene [ C19H38, CAS ID=1000192-60-5 ] |
|  | 1-Decene, 2,4-dimethyl- [ C12H24, CAS ID=55170-80-4 ] |
|  | 1-Docosene [ C22H44, CAS ID=1599-67-3 ] |
|  | 1-Nonadecene [ C19H38, CAS ID=18435-45-5 ] |
|  | 1-Octene, 3,7-dimethyl- [ C10H20, CAS ID=4984-01-4 ] |
|  | 2,6-Dimethyldecane [ C12H26, CAS ID=13150-81-7 ] |
|  | 2-Decene, 7-methyl-, (Z)- [ C11H22, CAS ID=74630-23-2 ] |
|  | 2-methyloctacosane [ C29H60, CAS ID=1000376-72-8 ] |
|  | 2-methyltetracosane [ C25H52, CAS ID=1000376-72-6 ] |
|  | 3-Eicosene, (E)- [ C20H40, CAS ID=74685-33-9 ] |
|  | 3-Ethyl-3-methylheptane [ C10H22, CAS ID=17302-01-1 ] |
|  | Decane, 2,3,5,8-tetramethyl- [ C14H30, CAS ID=192823-15-7 ] |
|  | Decane, 2,3,5-trimethyl- [ C13H28, CAS ID=62238-11-3 ] |
|  | Decane, 3,3,5-trimethyl- [ C13H28, CAS ID=62338-13-0 ] |
|  | Decane, 3,3,6-trimethyl- [ C13H28, CAS ID=62338-14-1 ] |
|  | Decane, 3,3,8-trimethyl- [ C13H28, CAS ID=62338-16-3 ] |
|  | Decane, 3,8-dimethyl- [ C12H26, CAS ID=17312-55-9 ] |
|  | Decane, 4-methyl- [ C11H24, CAS ID=2847-72-5 ] |
|  | Decane, 5-ethyl-5-methyl- [ C13H28, CAS ID=17312-74-2 ] |
|  | Dodecane [ C12H26, CAS ID=112-40-3 ] |
|  | Dodecane, 2,6,10-trimethyl- [ C15H32, CAS ID=3891-98-3 ] |
|  | Dodecane, 2,6,11-trimethyl- [ C15H32, CAS ID=31295-56-4 ] |
|  | Dodecane, 2,7,10-trimethyl- [ C15H32, CAS ID=74645-98-0 ] |
|  | Dodecane, 4,6-dimethyl- [ C14H30, CAS ID=61141-72-8 ] |
|  | Eicosane [ C20H42, CAS ID=112-95-8 ] |
|  | Heneicosane [ C21H44, CAS ID=629-94-7 ] |
|  | Heptacosane [ C27H56, CAS ID=593-49-7 ] |
|  | Heptadecane, 2-methyl- [ C18H38, CAS ID=1560-89-0 ] |
|  | Heptadecane, 8-methyl- [ C18H38, CAS ID=13287-23-5 ] |
|  | Heptane, 2,3-dimethyl- [ C9H20, CAS ID=3074-71-3 ] |
|  | Heptane, 2,4-dimethyl- [ C9H20, CAS ID=2213-23-2 ] |
|  | Hexacosane [ C26H54, CAS ID=630-01-3 ] |
|  | Hexadecane [ C16H34, CAS ID=544-76-3 ] |
|  | Hexadecane, 2,6,10,14-tetramethyl- [ C20H42, CAS ID=638-36-8 ] |
|  | Hexadecane, 2,6,11,15-tetramethyl- [ C20H42, CAS ID=504-44-9 ] |
|  | Hexadecane, 2-methyl- [ C17H36, CAS ID=1560-92-5 ] |
|  | Hexadecane, 7-methyl- [ C17H36, CAS ID=26730-20-1 ] |
|  | Nonadecane [ C19H40, CAS ID=629-92-5 ] |
|  | Nonane [ C9H20, CAS ID=111-84-2 ] |
|  | Nonane, 2,6-dimethyl- [ C11H24, CAS ID=17302-28-2 ] |
|  | Nonane, 2-methyl-3-methylene- [ C11H22, CAS ID=55499-08-6 ] |
|  | Nonane, 3-methyl-5-propyl- [ C13H28, CAS ID=31081-18-2 ] |
|  | Nonane, 4,5-dimethyl- [ C11H24, CAS ID=17302-23-7 ] |
|  | Nonane, 5-(2-methylpropyl)- [ C13H28, CAS ID=62185-53-9 ] |
|  | Nonane, 5-methyl-5-propyl- [ C13H28, CAS ID=17312-75-3 ] |
|  | Octacosane [ C28H58, CAS ID=630-02-4 ] |
|  | Octadecane [ C18H38, CAS ID=593-45-3 ] |
|  | Octane, 2,3,6,7-tetramethyl- [ C12H26, CAS ID=52670-34-5 ] |
|  | Octane, 4-methyl- [ C9H20, CAS ID=2216-34-4 ] |
|  | Octane, 5-ethyl-2-methyl- [ C11H24, CAS ID=62016-18-6 ] |
|  | Octane, 6-ethyl-2-methyl- [ C11H24, CAS ID=62016-19-7 ] |
|  | Pentadecane [ C15H32, CAS ID=629-62-9 ] |
|  | Pentadecane, 2,6,10-trimethyl- [ C18H38, CAS ID=3892-00-0 ] |
|  | Tetracosane [ C24H50, CAS ID=646-31-1 ] |
|  | Tetradecane, 2,6,10-trimethyl- [ C17H36, CAS ID=14905-56-7 ] |
|  | Tetradecane, 2-methyl- [ C15H32, CAS ID=1560-95-8 ] |
|  | Tetradecane, 4-methyl- [ C15H32, CAS ID=25117-24-2 ] |
|  | Tridecane [ C13H28, CAS ID=629-50-5 ] |
|  | Undecane, 2,10-dimethyl- [ C13H28, CAS ID=17301-27-8 ] |
|  | Undecane, 3,7-dimethyl- [ C13H28, CAS ID=17301-29-0 ] |
|  | Undecane, 3,8-dimethyl- [ C13H28, CAS ID=17301-30-3 ] |
|  | Undecane, 4,7-dimethyl- [ C13H28, CAS ID=17301-32-5 ] |
|  | Undecane, 4,8-dimethyl- [ C13H28, CAS ID=17301-33-6 ] |
|  | Undecane, 4-methyl- [ C12H26, CAS ID=2980-69-0 ] |
|  | Undecane, 5,7-dimethyl- [ C13H28, CAS ID=17312-83-3 ] |
|  | Undecane, 5-cyclohexyl- [ C17H34, CAS ID=13151-80-9 ] |
|  | 1-Dodecanol, 2-octyl- [ C20H42O, CAS ID=5333-42-6 ] |
|  | 4-Octene, 2,3,6-trimethyl- [ C11H22, CAS ID=63830-65-9 ] |
|  | 2,3-Dimethyldodecane [ C14H30, CAS ID=6117-98-2 ] |
|  | Hexane, 3,3,4,4-tetramethyl- [ C10H22, CAS ID=5171-84-6 ] |
|  | Tetradecane, 5-methyl- [ C15H32, CAS ID=25117-32-2 ] |
|  | (E,E)-7,11,15-Trimethyl-3-methylene-hexadeca-1,6,10,14-tetraene [ C20H32, CAS ID=70901-63-2 ] |
|  | Pentane, 3-ethyl-2-methyl- [ C8H18, CAS ID=609-26-7 ] |
|  | trans,trans-1,6-Dimethylspiro[4.5]decane [ C12H22, CAS ID=1000111-72-1 ] |
| Cyclic hydrocarbons | 1,3-Dioxane, 4,4-dimethyl- [ C6H12O2, CAS ID=766-15-4 ] |
|  | Cyclodecacyclotetradecene, 14,15-didehydro-1,4,5,8,9,10,11,12,13,16,17,18,19,20-tetradecahydro- [ C22H32, CAS ID=14113-61-2 ] |
|  | Cyclohexane, 1,2,3,4,5,6-hexaethyl- [ C18H36, CAS ID=1795-14-8 ] |
|  | Cyclohexane, 1-ethyl-2-propyl- [ C11H22, CAS ID=62238-33-9 ] |
|  | Cycloocta-1,3,6-triene, 2,3,5,5,8,8-hexamethyl- [ C14H22, CAS ID=1000161-97-9 ] |
|  | Cyclopentane, 1,1,3,3-tetramethyl- [ C9H18, CAS ID=50876-33-0 ] |
|  | Cyclopentane, 1-pentyl-2-propyl- [ C13H26, CAS ID=62199-51-3 ] |
|  | Cyclopropane, 1,1,2,3-tetramethyl- [ C7H14, CAS ID=74752-93-5 ] |
|  | Naphthalene [ C10H8, CAS ID=91-20-3 ] |
|  | Naphthalene, decahydro-2,3-dimethyl- [ C12H22, CAS ID=1008-80-6 ] |
|  | Tricyclo[4.2.2.0(2,5)]dec-7-ene, 7-(5-hexynyl)- [ C16H22, CAS ID=1000164-40-9 ] |
|  | Furan, tetrahydro-2,2,4,4-tetramethyl- [ C8H16O, CAS ID=3358-28-9 ] |
|  | Cyclohexane, (3,3-dimethylpentyl)- [ C13H26, CAS ID=61142-22-1 ] |
|  | Cyclooctane, butyl- [ C12H24, CAS ID=16538-93-5 ] |
|  | Dibutyl phthalate [ C16H22O4, CAS ID=84-74-2 ] |
|  | 5-Ethyl-1-nonene [ C11H22, CAS ID=19780-74-6 ] |
| Alcohol | 11-Methyldodecanol [ C13H28O, CAS ID=85763-57-1 ] |
|  | 1-Decanol, 2-hexyl- [ C16H34O, CAS ID=2425-77-6 ] |
|  | 1-Decanol, 2-methyl- [ C11H24O, CAS ID=18675-24-6 ] |
|  | 1-Dodecanol, 2-hexyl- [ C18H38O, CAS ID=110225-00-8 ] |
|  | 1-Heptanol, 2,4-diethyl- [ C11H24O, CAS ID=80192-55-8 ] |
|  | 1-Octanol, 2-butyl- [ C12H26O, CAS ID=3913-02-8 ] |
|  | 2,6,10,14-Hexadecatetraen-1-ol, 3,7,11,15-tetramethyl-, acetate, (E,E,E)- [ C22H36O2, CAS ID=61691-98-3 ] |
|  | 2,6-Octadien-1-ol, 3,7-dimethyl-, acetate, (Z)- [ C12H20O2, CAS ID=141-12-8 ] |
|  | 2-Hexyl-1-octanol [ C14H30O, CAS ID=19780-79-1 ] |
|  | 2-Isopropyl-5-methyl-1-heptanol [ C11H24O, CAS ID=91337-07-4 ] |
|  | 4-Hexen-1-ol, 5-methyl-2-(1-methylethenyl)-, acetate [ C12H20O2, CAS ID=25905-14-0 ] |
|  | Tridecanol, 2-ethyl-2-methyl- [ C16H34O, CAS ID=1000115-66-1 ] |
|  | 9-(3,3-Dimethyloxiran-2-yl)-2,7-dimethylnona-2,6-dien-1-ol [ C15H26O2, CAS ID=1000192-15-6 ] |
|  | Behenic alcohol [ C22H46O, CAS ID=661-19-8 ] |
| Aldehyde | Hexadecanal [ C16H32O, CAS ID=629-80-1 ] |
|  | Pentanal, 2,2-dimethyl- [ C7H14O, CAS ID=14250-88-5 ] |
| Ketones | 2-Heptanone, 3-methyl- [ C8H16O, CAS ID=2371-19-9 ] |
|  | Ethanone, 1-(3-ethyloxiranyl)- [ C6H10O2, CAS ID=17257-81-7 ] |
|  | 2-Cyclohexen-1-one, 4-(3-hydroxy-1-butenyl)-3,5,5-trimethyl- [ C13H20O2, CAS ID=34318-21-3 ] |
|  | Ethanone, 1-(3-methyloxiranyl)- [ C5H8O2, CAS ID=17257-79-3 ] |
|  | 3-Nonen-2-one, 3-ethyl- [ C11H20O, CAS ID=56312-56-2 ] |
|  | 4-Hexen-2-one [ C6H10O, CAS ID=25659-22-7 ] |
|  | 2-Hexanone, 5-methyl- [ C7H14O, CAS ID=110-12-3 ] |
| Acids | Oxalic acid, cyclohexyl propyl ester [ C11H18O4, CAS ID=1000309-30-3 ] |
|  | Oxalic acid, cyclohexyl pentyl ester [ C13H22O4, CAS ID=1000309-30-6 ] |
|  | Sulfurous acid, 2-ethylhexyl undecyl ester [ C19H40O3S, CAS ID=1000309-19-4 ] |
|  | 4,7,10,13,16,19-Docosahexaenoic acid, methyl ester, (all-Z)- [ C23H34O2, CAS ID=2566-90-7 ] |
|  | Sulfurous acid, pentyl undecyl ester [ C16H34O3S, CAS ID=1000309-14-4 ] |
|  | 1,2-Benzenedicarboxylic acid, bis(2-methylpropyl) ester [ C16H22O4, CAS ID=84-69-5 ] |
|  | Sulfurous acid, octadecyl 2-propyl ester [ C21H44O3S, CAS ID=1000309-12-7 ] |
|  | Sulfurous acid, 2-ethylhexyl isohexyl ester [ C14H30O3S, CAS ID=1000309-19-0 ] |
|  | Sulfurous acid, dodecyl 2-ethylhexyl ester [ C20H42O3S, CAS ID=1000309-19-5 ] |
|  | Sulfurous acid, 2-ethylhexyl tetradecyl ester [ C22H46O3S, CAS ID=1000309-19-7 ] |
|  | Sulfurous acid, 2-ethylhexyl hexyl ester [ C14H30O3S, CAS ID=1000309-20-2 ] |
|  | Sulfurous acid, pentadecyl 2-propyl ester [ C18H38O3S, CAS ID=1000309-12-6 ] |
|  | Sulfurous acid, hexyl pentadecyl ester [ C21H44O3S, CAS ID=1000309-13-7 ] |
|  | Sulfurous acid, 2-ethylhexyl nonyl ester [ C17H36O3S, CAS ID=1000309-19-2 ] |
|  | Phthalic acid, isobutyl 4-octyl ester [ C20H30O4, CAS ID=1000314-84-7 ] |
|  | Phthalic acid, 6-ethyl-3-octyl butyl ester [ C22H34O4, CAS ID=1000315-17-4 ] |
|  | Phthalic acid, hept-4-yl isobutyl ester [ C19H28O4, CAS ID=1000356-78-3 ] |
|  | Sulfurous acid, pentadecyl pentyl ester [ C20H42O3S, CAS ID=1000309-14-8 ] |
|  | Phthalic acid, cyclohexyl isohexyl ester [ C18H24O4, CAS ID=1000315-34-0 ] |
|  | Bicyclo[2.2.1]hept-2-ene, 1,7,7-trimethyl- [ C10H16, CAS ID=464-17-5 ] |
|  | Limonene [ C10H16, CAS ID=138-86-3 ] |
|  | D-Limonene [ C10H16, CAS ID=5989-27-5 ] |
|  | 1s,4R,7R,11R-1,3,4,7-Tetramethyltricyclo[5.3.1.0(4,11)]undec-2-en-8-one [ C15H22O, CAS ID=137235-42-8 ] |
| terpenoid | Solavetivone [ C15H22O, CAS ID=54878-25-0 ] |
|  | 1-Hydroxy-1,7-dimethyl-4-isopropyl-2,7-cyclodecadiene [ C15H26O, CAS ID=72120-50-4 ] |
|  | Bicyclo[4.3.0]nonane, 7-methylene-2,4,4-trimethyl-2-vinyl- [ C15H24, CAS ID=1000156-11-9 ] |
|  | Spiro[4.5]decan-7-one, 1,8-dimethyl-8,9-epoxy-4-isopropyl- [ C15H24O2, CAS ID=61050-91-7 ] |
|  | 4,8,13-Cyclotetradecatriene-1,3-diol, 1,5,9-trimethyl-12-(1-methylethyl)- [ C20H34O2, CAS ID=7220-78-2 ] |
|  | Andrographolide [ C20H30O5, CAS ID=5508-58-7 ] |
|  | Phytol, acetate [ C22H42O2, CAS ID=1000375-01-4 ] |
|  | Phytol [ C20H40O, CAS ID=150-86-7 ] |
|  | Thunbergol [ C20H34O, CAS ID=25269-17-4 ] |
|  | Oxirane, 2,2-dimethyl-3-(3,7,12,16,20-pentamethyl-3,7,11,15,19-heneicosapentaenyl)-, (all-E)- [ C30H50O, CAS ID=7200-26-2 ] |
|  | Ergost-5-en-3-ol, (3.beta.)- [ C28H48O, CAS ID=4651-51-8 ] |
|  | Stigmasterol [ C29H48O, CAS ID=83-48-7 ] |
|  | .beta.-Amyrin [ C30H50O, CAS ID=559-70-6 ] |
|  | Squalene [ C30H50, CAS ID=111-02-4 ] |
| Benzenes | Benzene, (1-butylheptyl)- [ C17H28, CAS ID=4537-15-9 ] |
|  | Benzene, 1,3-bis(1-formylethyl)- [ C12H14O2, CAS ID=1000160-34-1 ] |
| Phenylpropanoid | Dihydrocoumarin, 4,4,5,7,8-pentamethyl [ C14H18O2, CAS ID=39170-97-3 ] |
|  | Phenol, 3-cyclohexyl- [ C12H16O, CAS ID=1943-95-9 ] |
|  | Phenol, 2,4-bis(1,1-dimethylethyl)- [ C14H22O, CAS ID=96-76-4 ] |
|  | Benzaldehyde, 2,5-dimethyl- [ C9H10O, CAS ID=5779-94-2 ] |
|  | Benzaldehyde, 2,4-dimethyl- [ C9H10O, CAS ID=15764-16-6 ] |
|  | Phenol, 2,6-bis(1,1-dimethylethyl)-4-ethyl- [ C16H26O, CAS ID=4130-42-1 ] |
| N-containing natural products | Pyridine, 3-(1-methyl-2-pyrrolidinyl)-, (S)- [ C10H14N2, CAS ID=54-11-5 ] |
|  | 2-Piperidinone, N-[4-bromo-n-butyl]- [ C9H16BrNO, CAS ID=195194-80-0 ] |
|  | Tributylamine [ C12H27N, CAS ID=102-82-9 ] |
|  | N,4-Diethyl-4-heptanamine [ C11H25N, CAS ID=71275-07-5 ] |
| Other metabolites | 2-Bromo dodecane [ C12H25Br, CAS ID=13187-99-0 ] |
|  | Dodecane, 1-iodo- [ C12H25I, CAS ID=4292-19-7 ] |
|  | 2-Bromotetradecane [ C14H29Br, CAS ID=74036-95-6 ] |
|  | Decane, 1-iodo- [ C10H21I, CAS ID=2050-77-3 ] |
|  | Cyclopropane, 2-bromo-1,1,3-trimethyl- [ C6H11Br, CAS ID=36617-00-2 ] |
|  | Sulfurous acid, pentyl tetradecyl ester [ C19H40O3S, CAS ID=1000309-14-7 ] |
|  | Hexane, 2-bromo- [ C6H13Br, CAS ID=3377-86-4 ] |
