# Supplementary material for: Untargeted Metabolomics of Nicotiana tabacum Grown in United States and India Characterizes the Association of Plant Metabolomes With Natural Climate and Geography
Source: Front Plant Sci. 2019 Oct 30;10:1370. doi: 10.3389/fpls.2019.01370 (PMC6831618; doi:10.3389/fpls.2019.01370)
Supplement: Supplementary file 15 [file Table_8.docx]

Supplementary Table 8 Two hundred and twenty-five polar metabolites annotated from 366 samples

| Categories | Metabolites Annotated |
| --- | --- |
| Organic acid | Alpha-Linolenic acid, trimethylsilyl ester [ C21H38O2Si, CAS ID=97844-13-8 ] |
|  | Butanoic acid, 2,4-bis[(trimethylsilyl)oxy]-, trimethylsilyl ester [ C13H32O4Si3, CAS ID=55191-52-1 ] |
|  | 11-Eicosenoic acid, trimethylsilyl ester [ C23H46O2Si, CAS ID=1000079-04-0 ] |
|  | 1-Cyclohexene-1-carboxylic acid, 3,4,5-tris[(trimethylsilyl)oxy]-, trimethylsilyl ester, [3R-(3.alpha.,4.alpha.,5.beta.)]- [ C19H42O5Si4, CAS ID=55520-78-0 ] |
|  | Propanedioic acid, bis(trimethylsilyl) ester [ C9H20O4Si2, CAS ID=18457-04-0 ] |
|  | 2-Ethyl-3-hydroxypropionic acid, di-TMS [ C11H26O3Si2, CAS ID=1000072-00-1 ] |
|  | Butanoic acid, 3,4-bis[(trimethylsilyl)oxy]-, trimethylsilyl ester [ C13H32O4Si3, CAS ID=55191-53-2 ] |
|  | Butanoic acid, 4-[bis(trimethylsilyl)amino]-, trimethylsilyl ester [ C13H33NO2Si3, CAS ID=39508-23-1 ] |
|  | cis-13-Docosenoic acid, trimethylsilyl ester [ C25H50O2Si, CAS ID=1000333-24-2 ] |
|  | Eicosanoic acid, trimethylsilyl ester [ C23H48O2Si, CAS ID=55530-70-6 ] |
|  | Erythro-Pentonic acid, 2-deoxy-3,4,5-tris-O-(trimethylsilyl)-, trimethylsilyl ester [ C17H42O5Si4, CAS ID=74742-30-6 ]( C5H10O2) |
|  | Ethanedioic acid, bis(trimethylsilyl) ester [ C8H18O4Si2, CAS ID=18294-04-7 ] |
|  | Hexadecanoic acid, trimethylsilyl ester [ C19H40O2Si, CAS ID=55520-89-3 ] |
|  | L-(+)-Tartaric acid, bis(trimethylsilyl) ether, bis(trimethylsilyl) ester [ C16H38O6Si4, CAS ID=1000352-52-1 ] |
|  | Malonic acid, bis(2-trimethylsilylethyl ester [ C13H28O4Si2, CAS ID=90744-45-9 ] |
|  | Octadecanoic acid, trimethylsilyl ester [ C21H44O2Si, CAS ID=18748-91-9 ] |
|  | Pentonic acid, 5-deoxy-2,3-bis-O-(trimethylsilyl)-, .gamma.-lactone [ C11H24O4Si2, CAS ID=74742-33-9 ] |
|  | Phosphoric acid, bis(trimethylsilyl) 2,3-bis[(trimethylsilyl)oxy]propyl ester [ C15H41O6PSi4, CAS ID=31038-11-6 ] |
|  | Phosphoric acid, bis(trimethylsilyl)monomethyl ester [ C7H21O4PSi2, CAS ID=18291-81-1 ] |
|  | Propanedioic acid, [(trimethylsilyl)oxy]-, bis(trimethylsilyl) ester [ C12H28O5Si3, CAS ID=38165-93-4 ] |
|  | Tetradecanoic acid, trimethylsilyl ester [ C17H36O2Si, CAS ID=18603-17-3 ] |
|  | trans-9-Octadecenoic acid, trimethylsilyl ester [ C21H42O2Si, CAS ID=96851-47-7 ] |
|  | 2-Ketobutyric acid eo-tms [ C9H19NO3Si, CAS ID=1000332-24-7 ] |
|  | 1,2,3-Propanetricarboxylic acid, 2-[(trimethylsilyl)oxy]-, tris(trimethylsilyl) ester [ C18H40O7Si4, CAS ID=14330-97-3 ] |
|  | 2-Butenedioic acid (Z)-, bis(trimethylsilyl) ester [ C10H20O4Si2, CAS ID=23508-82-9 ] |
|  | Butanedioic acid, methyl-, bis(trimethylsilyl) ester [ C11H24O4Si2, CAS ID=55557-26-1 ] |
|  | Butanedioic acid, bis(trimethylsilyl) ester [ C10H22O4Si2, CAS ID=40309-57-7 ] |
|  | Propanoic acid, 2,3-bis[(trimethylsilyl)oxy]-, trimethylsilyl ester [ C12H30O4Si3, CAS ID=38191-87-6 ] |
|  | 2-Butenedioic acid (E)-, bis(trimethylsilyl) ester [ C10H20O4Si2, CAS ID=17962-03-7 ] |
|  | Propanoic acid, 3-[(trimethylsilyl)oxy]-, trimethylsilyl ester [ C9H22O3Si2, CAS ID=55162-32-8 ] |
|  | Benzoic acid trimethylsilyl ester [ C10H14O2Si, CAS ID=2078-12-8 ] |
|  | Benzoic acid, 3-[(trimethylsilyl)oxy]-, trimethylsilyl ester [ C13H22O3Si2, CAS ID=3782-84-1 ] |
|  | Benzoic acid, 2,6-bis(trimethylsiloxy)-, methyl ester [ C14H24O4Si2, CAS ID=27798-57-8 ] |
|  | Acetic acid, trifluoro-, 3,7-dimethyloctyl ester [ C12H21F3O2, CAS ID=28745-07-5 ] |
|  | Butanedioic acid, [(trimethylsilyl)oxy]-, bis(trimethylsilyl) ester [ C13H30O5Si3, CAS ID=38166-11-9 ] |
|  | Phosphoric acid, bis[2,3-bis[(trimethylsilyl)oxy]propyl] trimethylsilyl ester [ C21H55O8PSi5, CAS ID=32046-28-9 ] |
|  | 9,12-Octadecadienoic acid (Z,Z)-, trimethylsilyl ester [ C21H40O2Si, CAS ID=56259-07-5 ] |
|  | Propanoic acid, 2-[(trimethylsilyl)oxy]-, trimethylsilyl ester [ C9H22O3Si2, CAS ID=17596-96-2 ] |
|  | 2-Propenoic acid, 2-[(trimethylsilyl)oxy]-, trimethylsilyl ester [ C9H20O3Si2, CAS ID=55191-13-4 ] |
|  | Acetic acid, [(trimethylsilyl)oxy]-, trimethylsilyl ester [ C8H20O3Si2, CAS ID=33581-77-0 ] |
|  | 3,5-di-tert-Butyl-4-hydroxyphenylpropionic acid [ C17H26O3, CAS ID=20170-32-5 ] |
|  | 2,3,4-Trihydroxybutyric acid tetrakis(trimethylsilyl) deriv., (, (R*,R*)-) [ C16H40O5Si4, CAS ID=38191-88-7 ] |
| Sugar | d-Glucose, 2,3,4,5,6-pentakis-O-(trimethylsilyl)-, o-methyloxyme, (1Z)- [ C22H55NO6Si5, CAS ID=128705-73-7 ] |
|  | d-Mannose, 2,3,4,5,6-pentakis-O-(trimethylsilyl)-, o-methyloxyme, (1Z)- [ C22H55NO6Si5, CAS ID=128705-67-9 ] |
|  | .alpha.-D-Galactopyranose, 1,2,3,4,6-pentakis-O-(trimethylsilyl)- [ C21H52O6Si5, CAS ID=32166-80-6 ] |
|  | .alpha.-D-Glucopyranoside, 1,3,4,6-tetrakis-O-(trimethylsilyl)-.beta.-D-fructofuranosyl 2,3,4,6-tetrakis-O-(trimethylsilyl)- [ C36H86O11Si8, CAS ID=19159-25-2 ] |
|  | .alpha.-D-Mannopyranoside, methyl 2,3,4,6-tetrakis-O-(trimethylsilyl)- [ C19H46O6Si4, CAS ID=1769-06-8 ] |
|  | .beta.-D-(+)-Talopyranose, pentakis(trimethylsilyl) ether [ C21H52O6Si5, CAS ID=1000380-17-1 ] |
|  | .beta.-D-Allopyranose, pentakis(trimethylsilyl) ether [ C21H52O6Si5, CAS ID=1000380-16-2 ] |
|  | .beta.-D-Glucopyranose, 1,2,3,4,6-pentakis-O-(trimethylsilyl)- [ C21H52O6Si5, CAS ID=2775-90-8 ] |
|  | .beta.-d-Glucopyranoside, 2-[[4-(trimethylsilyl)oxy]phenyl]ethyl, tetrakis(trimethylsilyl) [ C29H60O7Si5, CAS ID=1000115-98-2 ] |
|  | .beta.-Gentiobiose, octakis(trimethylsilyl) ether [ C36H86O11Si8, CAS ID=1000380-09-2 ] |
|  | 2-.alpha.-Mannobiose, octakis(trimethylsilyl) ether (isomer 2) [ C36H86O11Si8, CAS ID=1000380-09-0 ] |
|  | 2-Deoxy-galactopyranose, tetrakis(trimethylsilyl) [ C18H44O5Si4, CAS ID=1000079-29-5 ] |
|  | 2-Keto-l-gluconic acid, penta(O-trimethylsilyl)- [ C21H50O7Si5, CAS ID=1000059-82-4 ] |
|  | 2-O-Glycerol-.alpha.-d-galactopyranoside, hexa-TMS [ C27H66O8Si6, CAS ID=1000098-09-0 ] |
|  | 3-.alpha.-Mannobiose, octakis(trimethylsilyl) ether (isomer 1) [ C36H86O11Si8, CAS ID=1000380-09-5 ] |
|  | 3-.alpha.-Mannobiose, octakis(trimethylsilyl) ether (isomer 2) [ C36H86O11Si8, CAS ID=1000380-09-6 ] |
|  | 4-O-.beta.-Galactopyranosyl-D-mannopyranose, octakis(trimethylsilyl) ether (isomer 2) [ C36H86O11Si8, CAS ID=1000380-08-8 ] |
|  | Adonitol, pentakis(trimethylsilyl) ether [ C20H52O5Si5, CAS ID=35-22-3 ] |
|  | Arabinitol, pentakis-O-(trimethylsilyl)- [ C20H52O5Si5, CAS ID=25138-28-7 ] |
|  | Aucubin, hexakis(trimethylsilyl) ether [ C33H70O9Si6, CAS ID=1000380-08-5 ] |
|  | D-(-)-Lyxofuranose, tetrakis(trimethylsilyl) ether [ C17H42O5Si4, CAS ID=1000380-20-3 ] |
|  | D-(-)-Ribofuranose, tetrakis(trimethylsilyl) ether (isomer 2) [ C17H42O5Si4, CAS ID=1000380-11-7 ] |
|  | D-(-)-Tagatofuranose, pentakis(trimethylsilyl) ether (isomer 2) [ C21H52O6Si5, CAS ID=1000380-12-6 ] |
|  | D-(+)-Arabitol, pentakis(trimethylsilyl) ether [ C20H52O5Si5, CAS ID=1000380-15-2 ] |
|  | D-(+)-Cellobiose, octakis(trimethylsilyl) ether (isomer 1) [ C36H86O11Si8, CAS ID=1000380-07-9 ] |
|  | D-(+)-Galacturonic acid, O-pentakis(trimethylsilyl) deriv. [ C21H50O7Si5, CAS ID=1000380-13-2 ] |
|  | D-(+)-Talofuranose, pentakis(trimethylsilyl) ether (isomer 1) [ C21H52O6Si5, CAS ID=1000380-17-3 ] |
|  | D-(+)-Turanose, octakis(trimethylsilyl) ether [ C36H86O11Si8, CAS ID=1000380-08-4 ] |
|  | D-Arabinonic acid, 2,3,5-tris-O-(trimethylsilyl)-, .gamma.-lactone [ C14H32O5Si3, CAS ID=32384-55-7 ] |
|  | D-Galactose, 2,3,4,5,6-pentakis-O-(trimethylsilyl)- [ C21H52O6Si5, CAS ID=6736-94-3 ] |
|  | D-Gluconic acid, 2,3,4,5,6-pentakis-O-(trimethylsilyl)-, trimethylsilyl ester [ C24H60O7Si6, CAS ID=34290-52-3 ] |
|  | D-Glucose, 4-O-[2,3,4,6-tetrakis-O-(trimethylsilyl)-.beta.-D-glucopyranosyl]-2,3,5,6-tetrakis-O-(trimethylsilyl)- [ C36H86O11Si8, CAS ID=56145-25-6 ] |
|  | D-Glucuronic acid, 2,3,4,5-tetrakis-O-(trimethylsilyl)-, trimethylsilyl ester [ C21H50O7Si5, CAS ID=55530-80-8 ] |
|  | D-Mannitol, 1,2,3,4,5,6-hexakis-O-(trimethylsilyl)- [ C24H62O6Si6, CAS ID=14317-07-8 ] |
|  | D-Psicofuranose, pentakis(trimethylsilyl) ether (isomer 2) [ C21H52O6Si5, CAS ID=1000380-12-8 ] |
|  | D-Ribose, 2,3,4,5-tetrakis-O-(trimethylsilyl)- [ C17H42O5Si4, CAS ID=33648-69-0 ] |
|  | D-Sorbitol, hexakis(trimethylsilyl) ether [ C24H62O6Si6, CAS ID=1000380-14-5 ] |
|  | Dulcitol, hexakis(trimethylsilyl) ether [ C24H62O6Si6, CAS ID=35-23-4 ] |
|  | D-Xylopyranose, 1,2,3,4-tetrakis-O-(trimethylsilyl)- [ C17H42O5Si4, CAS ID=55555-45-8 ] |
|  | D-Xylose, tetrakis(trimethylsilyl)- [ C17H42O5Si4, CAS ID=18623-22-8 ] |
|  | Ethyl 2,3,4,6-tetrakis-O-(trimethylsilyl)-D-glucopyranoside [ C20H48O6Si4, CAS ID=1000365-90-0 ] |
|  | Galactaric acid, 2,3,4,5-tetrakis-O-(trimethylsilyl)-, bis(trimethylsilyl) ester [ C24H58O8Si6, CAS ID=56272-61-8 ] |
|  | Galactinol, nonakis(trimethylsilyl) ether [ C39H94O11Si9, CAS ID=1000380-09-4 ] |
|  | Gluconic acid, 2-methoxime, tetra(trimethylsilyl)-, trimethylsilyl ester [ C22H53NO7Si5, CAS ID=1000062-87-7 ] |
|  | Glucopyranose, 1,2,3,4,6-pentakis-O-(trimethylsilyl)-, D- [ C21H52O6Si5, CAS ID=19126-99-9 ] |
|  | Glyceryl-glycoside TMS ether [ C27H66O8Si6, CAS ID=1000365-90-1 ] |
|  | Glycoside, .alpha.-methyl-trtrakis-O-(trimethylsilyl)- [ C19H46O6Si4, CAS ID=1000156-15-2 ] |
|  | Gulonic acid, 2,3,5,6-tetrakis-O-(trimethylsilyl)-, lactone [ C18H42O6Si4, CAS ID=55528-75-1 ] |
|  | L-(-)-Arabitol, pentakis(trimethylsilyl) ether [ C20H52O5Si5, CAS ID=35-21-2 ] |
|  | L-Threitol, tetrakis(trimethylsilyl) ether [ C16H42O4Si4, CAS ID=1000380-14-0 ] |
|  | Lyxose, tetra-(trimethylsilyl)-ether [ C17H42O5Si4, CAS ID=73745-85-4 ] |
|  | Maltose, octakis(trimethylsilyl)- [ C36H86O11Si8, CAS ID=33428-94-3 ] |
|  | Maltose, octakis(trimethylsilyl) ether (isomer 1) [ C36H86O11Si8, CAS ID=1000380-08-1 ] |
|  | Maltose, octakis(trimethylsilyl) ether (isomer 2) [ C36H86O11Si8, CAS ID=1000380-43-7 ] |
|  | Mannose, 6-deoxy-2,3,4,5-tetrakis-O-(trimethylsilyl)-, L- [ C18H44O5Si4, CAS ID=19127-15-2 ] |
|  | Melibiose, octakis(trimethylsilyl)- [ C36H86O11Si8, CAS ID=86087-85-6 ] |
|  | meso-Erythritol, tetrakis(trimethylsilyl) ether [ C16H42O4Si4, CAS ID=1000380-15-5 ] |
|  | Ribitol, 1,2,3,4,5-pentakis-O-(trimethylsilyl)- [ C20H52O5Si5, CAS ID=32381-53-6 ] |
|  | Ribonic acid, 2,3,4,5-tetrakis-O-(trimethylsilyl)-, trimethylsilyl ester [ C20H50O6Si5, CAS ID=57197-35-0 ] |
|  | Xylitol, 1,2,3,4,5-pentakis-O-(trimethylsilyl)- [ C20H52O5Si5, CAS ID=14199-72-5 ] |
|  | Xylonic acid, 2,3,4-tris-O-(trimethylsilyl)-, .delta.-lactone, D- [ C14H32O5Si3, CAS ID=32384-59-1 ] |
|  | .beta.-Gentiobiose, octakis(trimethylsilyl) ether, methyloxime (isomer 2) [ C37H89NO11Si8, CAS ID=1000380-11-0 ] |
|  | 2-.alpha.-Mannobiose, octakis(trimethylsilyl) ether, methyloxime (isomer 1) [ C37H89NO11Si8, CAS ID=1000380-10-7 ] |
|  | 2-.alpha.-Mannobiose, octakis(trimethylsilyl) ether, methyloxime (isomer 2) [ C37H89NO11Si8, CAS ID=1000380-10-8 ] |
|  | D-(-)-Fructose, pentakis(trimethylsilyl) ether, methyloxime (syn) [ C22H55NO6Si5, CAS ID=1000380-19-0 ] |
|  | D-(-)-Lyxose, tetrakis(trimethylsilyl) ether, methyloxime (syn) [ C18H45NO5Si4, CAS ID=1000380-18-6 ] |
|  | D-(-)-Tagatose, pentakis(trimethylsilyl) ether, trimethylsilyloxime (isomer 1) [ C24H61NO6Si6, CAS ID=1000380-38-7 ] |
|  | D-(+)-Cellobiose, octakis(trimethylsilyl) ether, methyloxime (isomer 1) [ C37H89NO11Si8, CAS ID=1000380-10-0 ] |
|  | D-(+)-Cellobiose, octakis(trimethylsilyl) ether, methyloxime (isomer 2) [ C37H89NO11Si8, CAS ID=1000380-09-9 ] |
|  | D-(+)-Galactose, pentakis(trimethylsilyl) ether, trimethylsilyloxime (isomer 2) [ C24H61NO6Si6, CAS ID=1000380-41-2 ] |
|  | D-(+)-Talose, pentakis(trimethylsilyl) ether, methyloxime (anti) [ C22H55NO6Si5, CAS ID=1000380-18-8 ] |
|  | D-(+)-Xylose, tetrakis(trimethylsilyl) ether, methyloxime (anti) [ C18H45NO5Si4, CAS ID=1000380-18-3 ] |
|  | D-(+)-Xylose, tetrakis(trimethylsilyl) ether, methyloxime (syn) [ C18H45NO5Si4, CAS ID=1000380-18-4 ] |
|  | D-Allose, pentakis(trimethylsilyl) ether, methyloxime (anti) [ C22H55NO6Si5, CAS ID=1000380-19-8 ] |
|  | D-Allose, pentakis(trimethylsilyl) ether, methyloxime (syn) [ C22H55NO6Si5, CAS ID=1000380-19-7 ] |
|  | D-Allose, pentakis(trimethylsilyl) ether, trimethylsilyloxime (isomer 1) [ C24H61NO6Si6, CAS ID=1000380-40-9 ] |
|  | D-Allose, pentakis(trimethylsilyl) ether, trimethylsilyloxime (isomer 2) [ C24H61NO6Si6, CAS ID=1000380-41-0 ] |
|  | D-Fructose, 1,3,4,5,6-pentakis-O-(trimethylsilyl)-, O-methyloxime [ C22H55NO6Si5, CAS ID=56196-14-6 ] |
|  | d-Galactose, 2,3,4,5,6-pentakis-O-(trimethylsilyl)-, o-methyloxyme, (1E)- [ C22H55NO6Si5, CAS ID=128705-64-6 ] |
|  | d-Galactose, 2,3,4,5,6-pentakis-O-(trimethylsilyl)-, o-methyloxyme, (1Z)- [ C22H55NO6Si5, CAS ID=128705-71-5 ] |
|  | D-Glucose, 2,3,4,5,6-pentakis-O-(trimethylsilyl)-, O-methyloxime [ C22H55NO6Si5, CAS ID=34152-44-8 ] |
|  | d-Glucose, 2,3,4,5,6-pentakis-O-(trimethylsilyl)-, o-methyloxyme, (1E)- [ C22H55NO6Si5, CAS ID=130405-10-6 ] |
|  | D-Lactose, octakis(trimethylsilyl) ether, methyloxime (isomer 2) [ C37H89NO11Si8, CAS ID=1000380-10-4 ] |
|  | DL-Arabinose, tetrakis(trimethylsilyl) ether, methyloxime [ C18H45NO5Si4, CAS ID=1000380-18-2 ] |
|  | d-Ribose, 2,3,4,5-tetrakis-O-(trimethylsilyl)-, O-methyloxime [ C18H45NO5Si4, CAS ID=56196-08-8 ] |
|  | Galactose oxime hexakis(trimethylsilyl) [ C24H61NO6Si6, CAS ID=120850-88-6 ] |
|  | Glucopyranose, pentakis-O-trimethylsilyl- [ C21H52O6Si5, CAS ID=1000157-49-7 ] |
|  | Gulose, 2,3,4,5,6-pentakis-O-(trimethylsilyl)- [ C21H52O6Si5, CAS ID=56192-86-0 ] |
|  | L-(+)-Threose, tris(trimethylsilyl) ether, trimethylsilyloxime (isomer 1) [ C16H41NO4Si4, CAS ID=1000380-40-4 ] |
|  | Lactulose, octakis(trimethylsilyl) ether, methyloxime (isomer 1) [ C37H89NO11Si8, CAS ID=1000380-09-7 ] |
|  | Maltose, octakis(trimethylsilyl) ether, methyloxime (isomer 1) [ C37H89NO11Si8, CAS ID=1000380-10-1 ] |
|  | Maltose, octakis(trimethylsilyl) ether, methyloxime (isomer 2) [ C37H89NO11Si8, CAS ID=1000380-10-2 ] |
|  | Palatinose, heptakis(trimethylsilyl) ether, methyloxime [ C33H79NO10Si7, CAS ID=1000380-11-1 ] |
|  | Trimethylsilyl ether of glucitol [ C24H62O6Si6, CAS ID=14199-80-5 ] |
|  | D-Mannose, 2,3,4,5-tetrakis-O-(trimethylsilyl)-, O-methyloxime, 6-[bis(trimethylsilyl) phosphate] [ C25H64NO9PSi6, CAS ID=55530-76-2 ] |
|  | Glucaric acid, 2,3,4,5-tetrakis-O-(trimethylsilyl)-, bis(trimethylsilyl) ester [ C24H58O8Si6, CAS ID=38165-96-7 ] |
|  | Arabinofuranose, 1,2,3,5-tetrakis-O-(trimethylsilyl)- [ C17H42O5Si4, CAS ID=55399-49-0 ] |
|  | D-Lactose, octakis(trimethylsilyl) ether (isomer 1) [ C36H86O11Si8, CAS ID=1000380-08-2 ] |
|  | L-Sorbose, pentakis(trimethylsilyl) ether, trimethylsilyloxime (isomer 1) [ C24H61NO6Si6, CAS ID=1000380-38-3 ] |
|  | Xylulose tetrakis(trimethylsilyl)- [ C17H42O5Si4, CAS ID=1000141-49-4 ] |
|  | Myo-Inositol, 1,2,3,4,5,6-hexakis-O-(trimethylsilyl)- [ C24H60O6Si6, CAS ID=2582-79-8 ] |
|  | Myo-Inositol, 1,3,4,5,6-pentakis-O-(trimethylsilyl)-, bis(trimethylsilyl) phosphate [ C27H69O9PSi7, CAS ID=33910-06-4 ] |
| Hydrocarbons | 1-Nonene, 4,6,8-trimethyl- [ C12H24, CAS ID=54410-98-9 ] |
|  | 1-Undecene, 7-methyl- [ C12H24, CAS ID=74630-42-5 ] |
|  | 2-(4-Methoxyphenyl)-2-(4-trimethoxysilyloxy)propane [ C19H26O2Si, CAS ID=1000283-55-5 ] |
|  | 2H,8H-Benzo[1,2-b:5,4-b']dipyran-10-propanol, 5-methoxy-2,2,8,8-tetramethyl- [ C20H26O4, CAS ID=26535-37-5 ] |
|  | 3,8-Dioxa-2,9-disiladecane, 2,2,9,9-tetramethyl- [ C10H26O2Si2, CAS ID=18001-91-7 ] |
|  | 3-Methyl-1,3-bis(trimethylsilyloxy)butane [ C11H28O2Si2, CAS ID=1000079-15-2 ] |
|  | 2-Decene, 7-methyl-, (Z)- [ C11H22, CAS ID=74630-23-2 ] |
|  | Disiloxane, hexamethyl- [ C6H18OSi2, CAS ID=107-46-0 ] |
|  | Dodecane, 4,6-dimethyl- [ C14H30, CAS ID=61141-72-8 ] |
|  | Pentasiloxane, dodecamethyl- [ C12H36O4Si5, CAS ID=141-63-9 ] |
|  | Silane, dimethyl(octadecyloxy)propyl- [ C23H50OSi, CAS ID=65597-99-1 ] |
|  | Trimethyl(2,6 ditert.-butylphenoxy)silane [ C17H30OSi, CAS ID=10416-73-6 ] |
|  | 1,1,1-Tris(hydroxymethyl)propane, tris(trimethylsilyl) ether [ C15H38O3Si3, CAS ID=1000352-39-8 ] |
|  | Benzene, (1-pentylheptyl)- [ C18H30, CAS ID=2719-62-2 ] |
|  | Benzene, 1,3-bis(1-formylethyl)- [ C12H14O2, CAS ID=1000160-34-1 ] |
|  | 1-Cyclohexyldimethylsilyloxyoctadecane [ C26H54OSi, CAS ID=1000281-96-8 ] |
|  | 3,8-Dioxa-2,9-disiladecane, 2,2,9,9-tetramethyl-5,6-bis[(trimethylsilyl)oxy]-, (R*,S*)- [ C16H42O4Si4, CAS ID=25258-02-0 ] |
|  | Tetrasiloxane, decamethyl- [ C10H30O3Si4, CAS ID=141-62-8 ] |
|  | Benzene, (1-butyloctyl)- [ C18H30, CAS ID=2719-63-3 ] |
|  | Cyclooctane, 1,4-dimethyl-, cis- [ C10H20, CAS ID=13151-99-0 ] |
|  | Trisiloxane, 1,1,1,5,5,5-hexamethyl-3,3-bis[(trimethylsilyl)oxy]- [ C12H36O4Si5, CAS ID=3555-47-3 ] |
|  | 4-Ethyl-4-methyl-1-hexene [ C9H18, CAS ID=90674-67-2 ] |
| Alcohols and ketone | Threitol, 1,2,3,4-tetrakis-O-(trimethylsilyl)-, D- [ C16H42O4Si4, CAS ID=32381-52-5 ] |
|  | Dithioerythritol, O,O',S,S'-tetrakis(trimethylsilyl)- [ C16H42O2S2Si4, CAS ID=1000079-30-7 ] |
|  | Glycerol, tris(trimethylsilyl) ether [ C12H32O3Si3, CAS ID=6787-10-6 ] |
|  | Bisphenol C [ C17H20O2, CAS ID=79-97-0 ] |
|  | 2(3H)-Furanone, dihydro-3,4-bis[(trimethylsilyl)oxy]-, trans- [ C10H22O4Si2, CAS ID=55220-79-6 ] |
| Phenylpropanoid | Trimethylsilyl 3,4-bis(trimethylsiloxy)cinnamate [ C18H32O4Si3, CAS ID=10586-03-5 ] |
|  | Benzaldehyde, 2,3,4,5-tetramethyl- [ C11H14O, CAS ID=29344-95-4 ] |
|  | Scopolin, tetra(trimethylsilyl)- [ C28H50O9Si4, CAS ID=1000108-98-9 ] |
| N-containing compounds | 1,2,3,6-Tetrahydro-2,3'-bipyridine [ C10H12N2, CAS ID=2743-90-0 ] |
|  | 2H-Pyrrol-2-one, 1,5-dihydro-1-methyl- [ C5H7NO, CAS ID=13950-21-5 ] |
|  | 2-Piperidinecarboxylic acid, trimethylsilyl ester [ C9H19NO2Si, CAS ID=55887-52-0 ] |
|  | 3-Ethoxyacrylonitrile [ C5H7NO, CAS ID=61310-53-0 ] |
|  | 3-Pyridinecarboxylic acid, trimethylsilyl ester [ C9H13NO2Si, CAS ID=25436-37-7 ] |
|  | 9H-Purin-6-amine, N,9-bis(trimethylsilyl)- [ C11H21N5Si2, CAS ID=17995-04-9 ] |
|  | Cadaverine, N,N,N',N'-tetrakis(trimethylsilyl) [ C17H46N2Si4, CAS ID=65898-76-2 ] |
|  | Ethyl pipecolinate [ C8H15NO2, CAS ID=15862-72-3 ] |
|  | Methylamine, N-cyclopentylidene- [ C6H11N, CAS ID=10599-83-4 ] |
|  | N,O,O'-Tris-(trimethylsilyl)-6-hydroxy-2-aminohexanoic acid [ C15H37NO3Si3, CAS ID=66434-54-6 ] |
|  | n-Butylamine, N,N-bis(trimethylsilyl) [ C10H27NSi2, CAS ID=18394-04-2 ] |
|  | Nicotine, 1'-demethyl-, (.+/-.)- [ C9H12N2, CAS ID=5746-86-1 ] |
|  | Nornicotine [ C9H12N2, CAS ID=494-97-3 ] |
|  | Oxazole, 2,4-dimethyl- [ C5H7NO, CAS ID=7208-05-1 ] |
|  | Phenylamine, 3-(pyrrolidin-1-yl)- [ C10H14N2, CAS ID=1000316-72-5 ] |
|  | Phenylethanolamine triTMS [ C17H35NOSi3, CAS ID=68595-84-6 ] |
|  | Pyridine, 3-(1-methyl-2-pyrrolidinyl)-, (S)- [ C10H14N2, CAS ID=54-11-5 ] |
|  | Pyridine, 3-trimethylsiloxy- [ C8H13NOSi, CAS ID=41571-88-4 ] |
|  | Silanamine, 1,1,1-trimethyl-N-(trimethylsilyl)-N-[2-[(trimethylsilyl)oxy]ethyl]- [ C11H31NOSi3, CAS ID=5630-81-9 ] |
|  | 1,4-Butanediamine, N,N,N',N'-tetrakis(trimethylsilyl)- [ C16H44N2Si4, CAS ID=39772-63-9 ] |
|  | Silanamine, 1,1,1-trimethyl-N-(trimethylsilyl)-N-[2-[(trimethylsilyl)oxy]-2-[4-[(trimethylsilyl)oxy]phenyl]ethyl]- [ C20H43NO2Si4, CAS ID=55556-99-5 ] |
|  | 2,5-Dimethylbenzonitrile [ C9H9N, CAS ID=13730-09-1 ] |
|  | Silanamine, N-[2-[2-methoxy-4-[(trimethylsilyl)oxy]phenyl]ethyl]-1,1,1-trimethyl-N-(trimethylsilyl)- [ C18H37NO2Si3, CAS ID=55530-69-3 ] |
|  | Butanal, 2,3,4-tris[(trimethylsilyl)oxy]-3-[[(trimethylsilyl)oxy]methyl]-, O-methyloxime, (S)- [ C18H45NO5Si4, CAS ID=56196-22-6 ] |
|  | 1,1,1,3,5,5,7,7,7-Nonamethyl-3-(trimethylsiloxy)tetrasiloxane [ C12H36O4Si5, CAS ID=38146-99-5 ] |
| Amino acid | Alanine, N-methyl-n-butoxycarbonyl-, octadecyl ester [ C27H53NO4, CAS ID=1000329-38-2 ] |
|  | Alanine, phenyl-, trimethylsilyl ester, dl- [ C12H19NO2Si, CAS ID=2899-42-5 ] |
|  | d-Proline, N-allyloxycarbonyl-, allyl ester [ C12H17NO4, CAS ID=1000320-97-8 ] |
|  | Glycine, N,N-bis(trimethylsilyl)-, trimethylsilyl ester [ C11H29NO2Si3, CAS ID=5630-82-0 ] |
|  | l-Glutamine, tris(trimethylsilyl) deriv. [ C14H34N2O3Si3, CAS ID=70591-28-5 ] |
|  | l-Leucine, trimethylsilyl ester [ C9H21NO2Si, CAS ID=1000333-25-0 ] |
|  | L-Proline, 1-(trimethylsilyl)-, trimethylsilyl ester [ C11H25NO2Si2, CAS ID=7364-47-8 ] |
|  | L-Serine, N,O-bis(trimethylsilyl)-, trimethylsilyl ester [ C12H31NO3Si3, CAS ID=7364-48-9 ] |
|  | L-Tryptophan, N,1-bis(trimethylsilyl)-, trimethylsilyl ester [ C20H36N2O2Si3, CAS ID=55429-28-2 ] |
|  | L-Tyrosine, N,O-bis(trimethylsilyl)-, trimethylsilyl ester [ C18H35NO3Si3, CAS ID=51220-73-6 ] |
|  | N,O,O-Tris(trimethylsilyl)-L-threonine [ C13H33NO3Si3, CAS ID=7537-02-2 ] |
|  | N,O-Bis-(trimethylsilyl)phenylalanine [ C15H27NO2Si2, CAS ID=2899-52-7 ] |
|  | Glutamic acid, N-(trimethylsilyl)-, bis(trimethylsilyl) ester, L- [ C14H33NO4Si3, CAS ID=15985-07-6 ] |
|  | L-Threonic acid, tris(trimethylsilyl) ether, trimethylsilyl ester [ C16H40O5Si4, CAS ID=1000352-53-3 ] |
|  | L-Proline, 5-oxo-1-(trimethylsilyl)-, trimethylsilyl ester [ C11H23NO3Si2, CAS ID=30274-77-2 ] |
|  | Serine, N,O-bis(trimethylsilyl)-, trimethylsilyl ester [ C12H31NO3Si3, CAS ID=64625-17-8 ] |
|  | N,O-Bis(trimethylsilyl)-L-phenylalanine [ C15H27NO2Si2, CAS ID=7364-51-4 ] |
|  | N,O-Bis-(trimethylsilyl)alanine [ C9H23NO2Si2, CAS ID=2899-44-7 ] |
|  | l-Aspartic acid, bis(trimethylsilyl) ester [ C10H23NO4Si2, CAS ID=1000333-28-8 ] |
|  | .beta.-Alanine, N,N-bis(trimethylsilyl)-, trimethylsilyl ester [ C12H31NO2Si3, CAS ID=55255-77-1 ] |
|  | Serine, bis(trimethylsilyl)- [ C9H23NO3Si2, CAS ID=70125-39-2 ] |
|  | l-Threonine, O-(trimethylsilyl)-, trimethylsilyl ester [ C10H25NO3Si2, CAS ID=7536-82-5 ] |
|  | L-Aspartic acid, N-(trimethylsilyl)-, bis(trimethylsilyl) ester [ C13H31NO4Si3, CAS ID=55268-53-6 ] |
|  | L-Asparagine, N,N2-bis(trimethylsilyl)-, trimethylsilyl ester [ C13H32N2O3Si3, CAS ID=55649-62-2 ] |
|  | l-Proline, trimethylsilyl ester [ C8H17NO2Si, CAS ID=1000333-26-2 ] |
|  | N,O,O'-Tris-(trimethylsilyl)threonine [ C13H33NO3Si3, CAS ID=64569-35-3 ] |
