# Supplementary material for: Untargeted Metabolomics of Nicotiana tabacum Grown in United States and India Characterizes the Association of Plant Metabolomes With Natural Climate and Geography
Source: Front Plant Sci. 2019 Oct 30;10:1370. doi: 10.3389/fpls.2019.01370 (PMC6831618; doi:10.3389/fpls.2019.01370)
Supplement: Supplementary file 16 [file Table_9.docx]

Supplementary Table 9 Metabolites detected in all two-year’s samples of plants in both North Carolina and India are grouped to compare their dynamic trends.

| Groups | Name of metabolites |
| --- | --- |
| Mono-terpenoid(4) | L-Limonene (C10H16) |
|  | Bicyclo[2.2.1]hept-2-ene, 1,7,7-trimethyl- (C10H16) |
|  | D-Limonene (C10H16) |
|  | Cyclopropane, 1,2-dimethyl-3-pentyl-( C10H20) |
| sesquiterpenoid(6) | Bicyclo[4.3.0]nonane, 7-methylene-2,4,4-trimethyl-2-vinyl- (C15H24) |
|  | 9-(3,3-Dimethyloxiran-2-yl)-2,7-dimethylnona-2,6-dien-1-ol (C15H26O2) |
|  | Solavetivone (C15H22O) |
|  | Spiro[4.5]decan-7-one, 1,8-dimethyl-8,9-epoxy-4-isopropyl-(C15H24O2) |
|  | 1-Hydroxy-1,7-dimethyl-4-isopropyl-2,7-cyclodecadiene (C15H26O) |
|  | 1s,4R,7R,11R-1,3,4,7-Tetramethyltricyclo[5.3.1.0(4,11)]undec-2-en-8-one (C15H22O) |
| Diterpenoids (6) | Phytol (C20H40O) |
|  | Andrographolide(C20H30O5) |
|  | Thunbergol (C20H34O) |
|  | (E,E)-7,11,15-Trimethyl-3-methylene-hexadeca-1,6,10,14-tetraene (C20H32) |
|  | 4,8,13-Cyclotetradecatriene-1,3-diol, 1,5,9-trimethyl-12-(1-methylethyl)- (C20H34O2) |
|  | Phytol, acetate (C22H42O2) |
| Triterpenoid(5) | beta-Amyrin (C30H50O) |
|  | Squalene (C30H50) |
|  | Stigmasterol (C29H48O) |
|  | Vitamin E (C29H50O2) |
|  | Ergost-5-en-3-ol, (3.beta.)- (C28H48O) |
| Nicotine | Nicotine |
| Benzenes (3) | Benzene, (1-butylheptyl)- (C17H28) |
|  | Benzene, (1-pentylheptyl)- (C18H30) |
|  | Benzene, 1,3-bis(1-formylethyl)- (C12H14O2) |
| PAH (2) | Naphthalene (C10H8) |
|  | Naphthalene, decahydro-2,3-dimethyl- (C12H22) |
| Amino acid (13) | Alanine |
|  | Glumamic acid |
|  | Glycine |
|  | Asparagine |
|  | Aspartic acid |
|  | Glutamine |
|  | Leucine |
|  | Proline |
|  | threonine |
|  | Tryptophan |
|  | Tyrosine |
|  | phenylalanine |
|  | Serine |
| Organic acid In TCA (3) | Citric acid |
|  | Fumaric acid |
|  | Succinic acid |
| Sugar (9) | Galactose |
|  | Glucose |
|  | Galactinol |
|  | Fructose |
|  | Xylose |
|  | Maltose |
|  | Mannose |
|  | Arabinose |
|  | Sorbitol |
| Nitrogen-containing secondary metabolites (5) | Nornicotine |
|  | Nicotine, 1'-demethyl |
|  | 1,2,3,6-Tetrahydro-2,3'-bipyridine |
|  | Phenylamine, 3-(pyrrolidin-1-yl)- |
|  | 3-hydroxypyridine |
| polyphenol (2) | cinnamate |
|  | Scopolin |
